# A bipartite NLS motif mediates the nuclear import of *Drosophila* moesin

**DOI:** 10.3389/fcell.2024.1206067

**Published:** 2024-02-21

**Authors:** Zoltán Kovács, Csaba Bajusz, Anikó Szabó, Péter Borkúti, Balázs Vedelek, Réka Benke, Zoltán Lipinszki, Ildikó Kristó, Péter Vilmos

**Affiliations:** ^1^ HUN-REN Biological Research Centre, Szeged, Hungary; ^2^ Doctoral School of Multidisciplinary Medical Science, University of Szeged, Szeged, Hungary; ^3^ HUN-REN Biological Research Centre, Institute of Biochemistry, MTA SZBK Lendület Laboratory of Cell Cycle Regulation, Szeged, Hungary

**Keywords:** *Drosophila*, moesin, ERM, nucleus, importin, phosphorylation, PIP2, NLS

## Abstract

The ERM protein family, which consists of three closely related proteins in vertebrates, ezrin, radixin, and moesin (ERM), is an ancient and important group of cytoplasmic actin-binding and organizing proteins. With their FERM domain, ERMs bind various transmembrane proteins and anchor them to the actin cortex through their C-terminal F-actin binding domain, thus they are major regulators of actin dynamics in the cell. ERMs participate in many fundamental cellular processes, such as phagocytosis, microvilli formation, T-cell activation and tumor metastasis. We have previously shown that, besides its cytoplasmic activities, the single ERM protein of *Drosophila melanogaster*, moesin, is also present in the cell nucleus, where it participates in gene expression and mRNA export. Here we study the mechanism by which moesin enters the nucleus. We show that the nuclear import of moesin is an NLS-mediated, active process. The nuclear localization sequence of the moesin protein is an evolutionarily highly conserved, conventional bipartite motif located on the surface of the FERM domain. Our experiments also reveal that the nuclear import of moesin does not require PIP2 binding or protein activation, and occurs in monomeric form. We propose, that the balance between the phosphorylated and non-phosphorylated protein pools determines the degree of nuclear import of moesin.

## 1 Introduction

In recent decades a remarkable discovery in cell biology has been that cytoskeletal proteins are also present in the nucleus (Kumeta et al., 2012; Percipalle and Vartiainen, 2019). The existence of a sophisticated molecular network in the nucleus, similar to the cytoskeleton is still in question today, but the results so far suggest that components of the cytoskeleton in the nucleus perform very diverse and important tasks (Kristó et al., 2016; Gunasekaran et al., 2022). Their quantities in the nucleus are tightly regulated (Kumeta et al., 2012), but with the exception of actin, surprisingly little is known about their nuclear transport mechanisms. The actin protein itself does not contain a canonical nuclear localization signal (NLS) sequence (Hofmann et al., 2009). It translocates into the nucleus in monomeric form by binding cofilin and Importin-9 (Dopie et al., 2012). The transport from the nucleus to the cytoplasm occurs via two leucine-rich export signals (NES) (Wada et al., 1998). In rat (Wada et al., 1998) and *Drosophila* (Collier et al., 2000), there are data that nuclear actin is exported by Exportin-1, which directly interacts with the NES signals. However, other works suggest that the NES motifs and Exportin-1 do not play a role in the nuclear export of actin, rather it is transported from the nucleus primarily in complex with profilin and Exportin-6 (Stüven et al., 2003; Bohnsack et al., 2006; Dopie et al., 2012).

One of the ancient and widespread groups of actin-binding cytoskeletal proteins in metazoans is the highly conserved ezrin-radixin-moesin (ERM) family of proteins. Ezrin, radixin and moesin are all present in vertebrates, whereas other invertebrate species only have one representative of the family (Shabardina et al., 2020). According to the current model, ERMs operate in the cytoplasm, where they anchor membrane proteins to the cortical actin network (Jankovics et al., 2002; Polesello et al., 2002), but they are also implicated in signal transduction as intermediaries in Rho signaling (Ivetic and Ridley, 2004). Thus, they play essential roles in basic cellular processes, such as cell-cell contact formation, signal transduction, cell migration (metastasis), phagocytosis, cell division, and apoptosis (Bosanquet et al., 2014; Michie et al., 2019; García-Ortiz and Serrador, 2020; Song et al., 2020). In our laboratory, we have previously shown that the only ERM protein of *Drosophila melanogaster*, *Drosophila* moesin (Moe), is also present in the cell nucleus (Kristó et al., 2016), where it regulates gene expression (Bajusz et al., 2021) and participates in mRNA export (Kristó et al., 2017).

Like other cytoskeletal proteins, the nuclear transport mechanisms of ERMs are not yet known. Their presence in the nucleus is indicated by only a few experimental data in the literature (Kaul et al., 1999; Bergquist et al., 2001; Melendez-Vasquez et al., 2001; Batchelor et al., 2004; Di Cristofano et al., 2010; Kristó et al., 2017). Some 20 years ago, Batchelor and co-workers identified an NLS motif in mammalian ERM proteins using cultured cells (Batchelor et al., 2004), and Krawetz and Kelly analyzed *in silico* the evolutionary conservation of predicted NLS motifs of ERMs in a few species (Krawetz and Kelly, 2008), but the import has not been studied in greater details to date. Therefore, we decided to define the NLS motif of *Drosophila* moesin, and examine its regulation and conservation.

## 2 Materials and methods

### 2.1 Generation of the transfection constructs

The mutant forms of moesin used in the experiments were generated with the QuickChange II Site Directed Mutagenesis Kit (Agilent, 200524) and the Q5 Site-Directed Mutagenesis Kit (New England BioLabs, E0554S). The T4 DNA polymerase used in the SLIC method was obtained from the New England BioLabs company (M0203S). The Moesin-pDONR221 vector was used as a template which contained the full length cDNA of *Drosophila* moesin and was created in our lab previously (Kristó et al., 2017). The DNA fragments encoding the different mutant moesin proteins were recombined into the pAWG vector using the Gateway LR Clonase II Enzyme Mix (Thermo Fisher Scientific, 11791-020), according to the manufacturer’s instructions. All constructs were sequenced before transfection. The MAL-GFP expression construct which contains the cDNA of the mouse MAL protein (Dopie et al., 2012) was a kind gift from Maria Vartiainen (University of Helsinki, Finland).

For the validation of the nuclear localization signal of moesin, the core moesin NLS sequence and surrounding amino acids (R274-T300) were amplified using the extNLS_F (CAA​GCA​CCG​GTC​CAC​TGG​CGG​CTC​CGG​CGG​CTC​CGG​CGG​CTC​CCG​TGT​CCG​CAT​CAA​CAA​GCG-3′) and sNLS_R (ATC​CTG​CTA​GCT​TAC​GTC​ACG​GTG​TCC​GGC​TTG​CGG​CGA​C-3′) primers which contain a sequence encoding the 3xGGS linker sequence for flexibility. The PCR product was cloned with InFusion cloning (Takara) into the *Drosophila* pAGW vector.

### 2.2 S2R+ cell maintenance, transfection, RNAi and drug treatment

The S2R+ *Drosophila* cell line (DGRC Stock 150; https://dgrc.bio.indiana.edu//stock/150; RRID:CVCL_Z831) was maintained at 25°C in Schneider’s *Drosophila* medium (Biowest, Cat.: L0207-500) complemented with 10% Fetal Bovine Serum (Fetal Bovine Serum, French Origin, Biowest, Cat.: S1820-500) and 1% antibiotics (Pen-Strep, Capricorn Scientific, Cat.: PS-B). To transfect the cells, the Effectene Transfection Reagent Kit (Qiagen, 301425) was used according to the manufacturer’s instructions. For live imaging, 8 × 10^5^ cells in 35 mm glass bottom Petri dishes (Cell E&G, GBD00001-200) were transfected with 500 ng of plasmid DNA, and grown for 2 days. For immunostaining, 1.5–2.0 × 10^5^ cells/well were transfected with 200–200 ng of each plasmid DNA, and grown for 5 days on 12 mm round glass cover slips (ROTH, P231.2) placed into the wells of 24-well cell culture dish (Thermo Fisher Scientific, Nunclon 24-Well × 1 mL MultiDish Cell Culture Dish, 142475).

For RNAi experiments, PCRs were performed on cDNA templates with target gene specific primers containing the T7 promoter sequence. To generate dsRNA, the PCR product was used in *in vitro* transcription assay (MEGAscript T7 transcription Kit, AM1334) according to the manufacturer’s instructions. Template DNA was digested, and the dsRNA was isolated (NucAway Spin Columns, AM10070). 200 ng dsRNA was added to each well.

Jasplakinolide desiccate (Invitrogen, Cat.: J7473) was dissolved in DMSO to create a stock solution of 1 mM. On the fifth day after transfection, cells were treated with Jasplakinolide in 5 μM final concentration for 2 hours prior to paraformaldehyde (PFA) fixation. As a control, cells were treated with equal amount of DMSO without Jasplakinolide. For Latrunculin A treatment cells were incubated for 20 min with either Latrunculin A (Sigma-Aldrich. L5163-100UG) at a final concentration of 5 μM, or an equal volume of its solvent DMSO (Sigma-Aldrich) as a control on the second day after transfection, and then fixed and immunostained.

### 2.3 Immunostaining of S2R+ cells

Transfected cells adhered to round glass coverslips were washed 1X with PBS, fixed in 4% PFA-PBS for 20 min at RT, then washed 3 × 2 minutes in PBS. Fixed cells were permeabilized with PBT (PBS +0.1% Triton X-100) for 5 min. Non-specific reactions were blocked with PBT-N solution (PBT, 1% BSA, 5% FCS) for 1 h. Samples were incubated overnight (O/N) with rabbit polyclonal anti-GFP (1:500, Thermo Fisher Scientific A-6455) primary antibody at 4°C. Next day the samples were washed 3 × 2 minutes with PBT and incubated with the fluorescently labeled secondary goat anti-rabbit Alexa Fluor 488 antibody (1:600, Thermo Fisher Scientific A-11008) for 1 h at RT in dark. After washing 3X with PBT, Phalloidin Alexa Fluor 546 (1:40, Thermo Fisher Scientific, A22283) and DAPI (0.2 μg/mL, Sigma-Aldrich) in PBS were applied for 2 h in dark, at RT. Samples were washed 3X in PBS, and the coverslips were placed upside down in a drop of mounting medium (Fluoromount G, Thermo Fisher Scientific, 00-4958-02) on a microscope slide. Images were taken with Olympus Fluoview FV1000 (×40 oil immersion objective, 1.3 NA), Zeiss LSM 800 and Leica TCS SP5 (×63 oil immersion objectives, 1.4 NA) confocal microscopes.

### 2.4 Quantification of nuclear accumulation and statistical analyses

The cells for quantification of nuclear/cytoplasmic levels were selected manually. Slides were first examined at low magnification to make sure the staining was uniform, then cells were selected for measurement by moving diagonally across the slide. The main criterion of selection was to be able to see a cytoplasm of sufficient size in the median plane of the nucleus. Cells with faint and very strong fluorescence were excluded from the analysis, but every other criterion (e.g., cell shape, cell size, nuclear volume) was ignored during the selection. With one exception (Figure 2C), DAPI staining was used to visualize the area of the nucleus and cytoplasm. The images showing DAPI staining were not included in the figures, but rather the nuclei were marked with arrows. For the cytoplasmic-nuclear fluorescence intensity measurements cells were measured once using ImageJ (Schneider et al., 2012). ROIs were drawn by hand in the entire nucleus and cytoplasm and the selected ROIs were used to measure pixel intensity values. In every measurement 25 cells per sample were examined, and every experiment was performed three times thus, the data of 3 × 25 cells were evaluated per sample in every experiment. Based on the normality of the data, either Student’s t-tests or the appropriate nonparametric Mann-Whitney U tests were performed for pairwise comparisons. In the case of Figure 3D, two-way ANOVA was performed to analyze the Rae1 and Slik RNAi effect on the nuclear and cytoplasmic distribution of GFP signal. Since the N/CP ratio values of Rae1 samples show non-normal distribution the Two-way ANOVA analysis was done by using the Aligned Rank Transformed (ART) (Wobbrock et al., 2011) N/CP ratio values. Graphs were created with GraphPad Prism 9.1.2 (GraphPad Software). Statistical significance is marked with ***p < 0.001, **p < 0.01, *p < 0.05, and *n.s.* (not significant).

In the assays we followed the method described previously for nuclear FRAP (Houtsmuller and Vermeulen, 2001; Day et al., 2012; Dopie et al., 2012), and the settings and parameters were used as described in (Dopie et al., 2012) with some modifications. In our experiment five pre-bleach frames were taken, then the nucleus was bleached with two to five frames. Pictures were taken every 1.317 s. A resolution of 512 × 512 pixel, and a scan speed of 400 Hz was applied. Transiently transfected S2R+ cells were imaged with a Leica TCS SP5 Confocal Microscope using a 63.0 × 1.40 oil immersion objective and the Leica LAS AF software with the FRAP Wizard module. Data represent three independent experiments and were analyzed with ImageJ, EasyFRAP and MS Excel software.

The FRAP curves for GFP and Moe-GFP were analyzed in Origin (8.1) by using one component exponential analysis and linear fitting. The averaged recovery curve of three measurements for GFP was fitted with the exponential decay equation y = y0 + A * EXP (−x/t) with fitted parameters y0 = 0.951 ± 0.0019, A = −0.6407 ± 0.0072 and t = 0.4033 ± 0.0088 min. The goodness of fit: Chi2∕DoF = 0.000256322 and R2 = 0.99079. The characteristic time of the slow processes can be calculated from the steepness of a linear fit based in the first derivate of the exponential function to be fit: y’ = A * EXP (-x/t) * (−1/t) and y’ (0) = −A/t. The parameters of the fitted a + b*x function: a = 0.44666 ± 0.00176 and b = 0.01865 ± 0.00182. R2 = 0.53815. The A (amplitude) parameter of an exponential decay can be read from the bleach depth: A = −0.550084552 ± 0.005381382 and using the error propagation, t = 29.49515023 ± 3.166893028 min.

### 2.5 Protein sequence alignments and 3D analysis

The ERM protein sequences analyzed for evolutionary conservation were obtained from the UniProt database (https://www.uniprot.org/) (UniProt Consortium, 2023) and are summarized in Table 1. For multiple sequence alignments Clustal Omega was used at EMBL-EBI (Madeira et al., 2022) with default settings.

**TABLE 1:** Summary of the ERM protein sequences analyzed for evolutionary conservation.

Species	Scientific name	UniProt ID
Human	*Homo sapiens*	P15311 (ezrin), P35241 (radixin), P26038 (moesin)
Mouse	*Mus musculus*	P26040 (ezrin), P26043 (radixin), P26041 (moesin)
Chick	*Gallus gallus*	A0A1D5NYK7 (moesin)
Clawed frog	*Xenopus laevis*	A0A1L8HAW1 (radixin), Q4V7Z2 (moesin)
Zebrafish	*Danio rerio*	Q5TZG5 (ezrin), Q66I42 (moesin)
Rice fish	*Oryzias latipes*	A0A0D6A9B1 (moesin)
Starfish	*Acanthaster planci*	A0A8B7ZPU5 (Radixin-like)
Fruit fly	*Drosophila melanogaster*	P46150 (moesin)
Red flour beetle	*Tribolium castaneum*	D6W9I6 (ERM1)
Silk moth	*Bombyx mori*	A0A8R1WN82 (ERM1)
Fall armyworm	*Spodoptera frugiperda*	A0T1L9 (ERM1)
Water bear	*Hypsibius dujardini*	A0A1W0WJ32 (ERM-like)
Roundworm1	*Caenorhabditis elegans*	G5EBK3 (ERM-1)
Roundworm2	*Caenorhabditis tropicalis*	A0A1I7V2T8 (ERM1)
Hydatid tapeworm	*Echinococcus granulosus*	W6UQS2 (ERM)
Hydra	*Hydra vulgaris*	T2MG47 (radixin)
Sponge	*Amphimedon queenslandica*	A0A1X7V0F6 (FERM domain containing)
Choanoflagellate	*Monosiga brevicollis*	A9URX5 (ERM-like)

For structural analysis data available at the RCSB Protein Data Bank (PDB) (http://www.rcsb.org/) (Berman et al., 2000) were used. To compose 3D protein structure and visualize the position of the NLS, the crystal structure of the insect *Spodoptera frugiperda* full-length moesin (PDB accession number 2I1K) (Li et al., 2007) was used, and modified with MolStar viewer (Mol*) (Sehnal et al., 2021).

## 3 Results

### 3.1 *Drosophila* moesin enters the nucleus by NLS-dependent, active nuclear import

**FIGURE 1:**
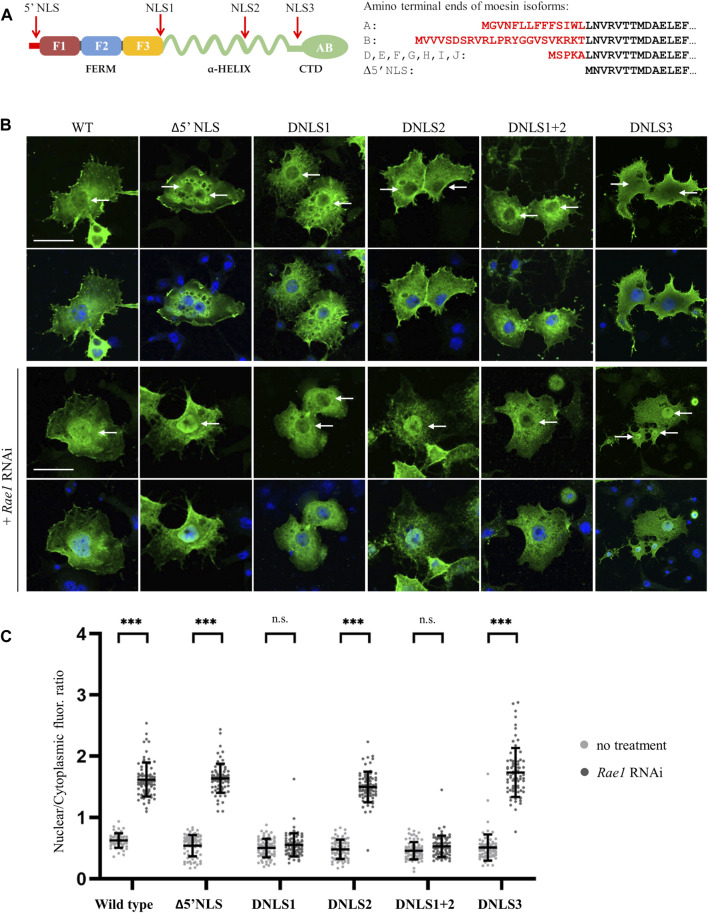
The nuclear transport of moesin is regulated by an NLS motif. **(A)** Positions of the three predicted nuclear localization signals (NLS1, NLS2, and NLS3) in the moesin protein and the sequence of the amino-terminal ends (5′NLS) in the different protein isoforms. Isoform C is truncated and, therefore, not shown here. F1-F3—subdomains of the FERM domain, CTD—C-Terminal Domain, AB—Actin Binding domain. **(B)** Representative images of *Drosophila* S2R+ cells expressing the putative NLS mutant forms of moesin (green) under normal condition and upon induction of nuclear import by *Rae1* silencing. Cells were stained for GFP; nuclei are visualized with DAPI staining (blue). Optical sections were obtained via confocal microscopy and one mid-plane is shown. Arrows point to nuclei. Scale bars: 25 μm **(C)** Quantification of the experiments in **(B)**. The graph shows the nuclear/cytoplasmic pixel intensity ratios, data represent mean rates +/− sd. Error bars represent standard deviation. Data represent mean ± SD of three independent experiments (*n* = 3), derived from the analysis of 25 cells per sample (3 × 25 in total per condition). Kolmogorov-Smirnov test was used to test for normality of data distribution. Untreated and *Rae1* RNAi treated samples were compared pairwise by Student’s *t*-test (DNLS1, DNLS2, DNLS1+2) or Mann-Whitney U test (wild type, Δ5′NLS, DNLS3). *p*-values: ***p < 0.001, and n.s. (not significant): *p* > 0.05.

The nuclear accumulation of *Drosophila* moesin upon transcriptional activation or after inhibition of mRNA export (Kristó et al., 2017) implies that the process is active and tightly regulated. To test this, first we investigated whether the protein contains a functional NLS motif. With the help of the NucPred (Brameier et al., 2007) and cNLS Mapper (Kosugi et al., 2009) software, we identified three potential NLS sites, the RRRK sequence at amino acids 294–297 (NLS1), the RRKQ motif between positions 447–450 (NLS2), and the GDAGG sequence at positions 485–489 (NLS3) (Figure 1A). Since *Drosophila* moesin protein isoforms differ mainly in their short N-terminal ends (Figure 1A) and, in the case of Merlin (moesin-ezrin-radixin-like-protein or NF2, the closest relative of ERMs), amino acids near the N-terminus are considered essential for nuclear translocation (Li et al., 2012), we also investigated the possibility of the amino-terminal end (5′NLS) being responsible for nuclear import (Figure 1A). Interestingly, despite the deletion of amino acids corresponding to NLS1, NLS2, NLS3 or in combination, as well as the deletion of the isoform-specific N-terminal ends (Δ5′NLS), moesin was still present in the nucleus (Figure 1B). As it is possible that, in accordance with our previous observations (Vilmos et al., 2009; Vilmos et al., 2016), the nuclear moesin we see in these experiments is incorporated during mitosis without any NLS-dependent import mechanism, we induced the nuclear import of moesin via inhibition of mRNA export by *Rae1* RNAi (Kristó et al., 2017) together with the expression of the different NLS mutant forms. We found that only the deletion of the NLS1 motif, hereafter referred to as Moe-DNLS, prevented moesin nuclear accumulation after mRNA export inhibition (Figure 1B, quantification in Figure 1C), revealing that NLS1 is a functional localization sequence and is responsible for the nuclear targeting of Moe.

**FIGURE 2:**
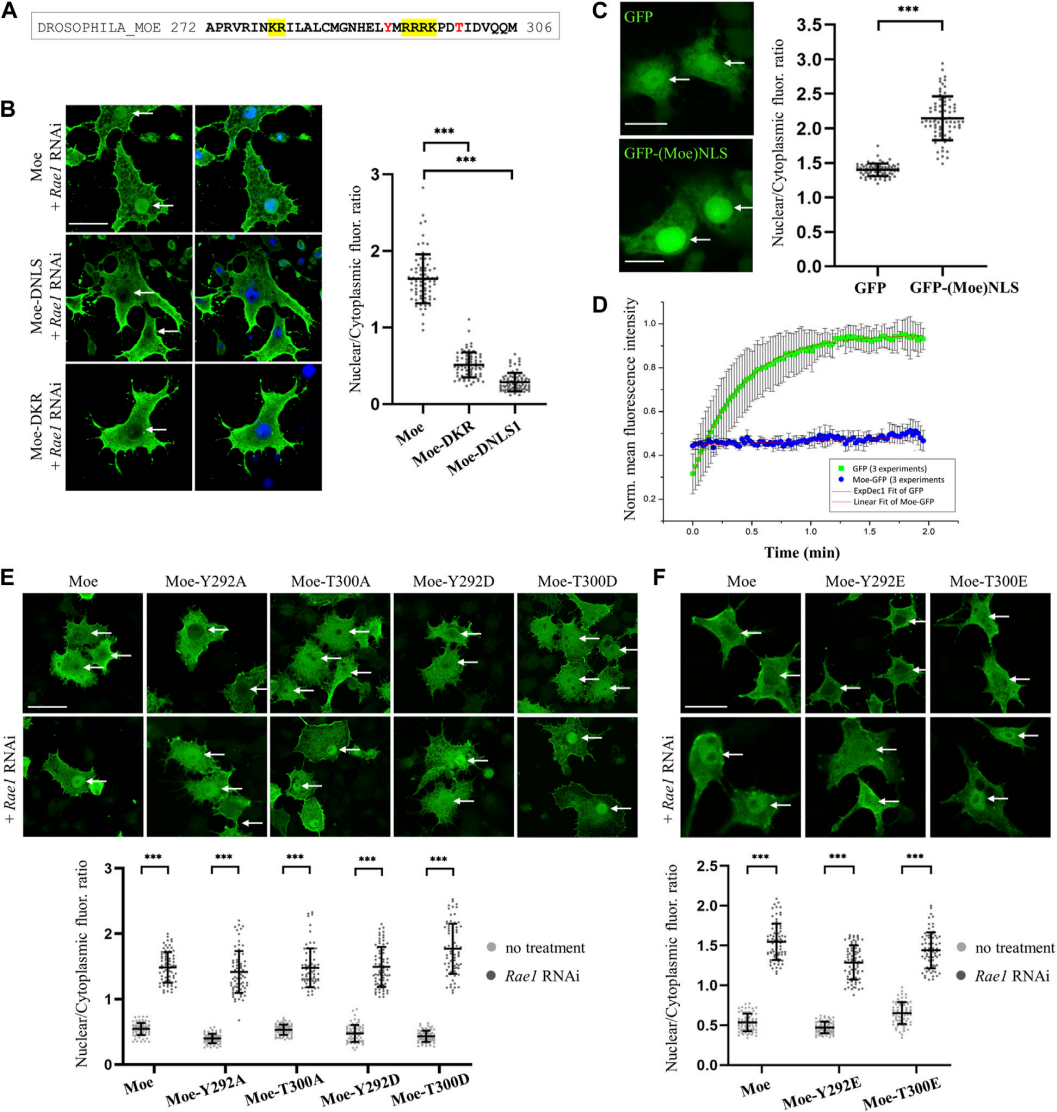
Analysis of the NLS motif. **(A)** Protein sequence in the vicinity of the NLS. The bipartite NLS is highlighted in yellow, conserved phosphorylatable residues are in red. **(B)** Anti-GFP antibody staining of S2R+ cells expressing the wild type, DNLS and the DKR_279-280_ mutant forms of moesin (green). Nuclear import was induced by *Rae1* RNAi. Nuclei are visualized with DAPI staining (blue). **(C)** The bipartite NLS motif of moesin directs the GFP protein (green) into the nucleus. Representative images of live S2R+ cells. **(D)** Nuclear import FRAP assay reveals that unlike the GFP protein, moesin is not freely diffusing into the nucleus. Data represent normalized mean nuclear to cytoplasmic fluorescence intensity ratios. +∕ − std (*n* = 3). Fitted (either exponential or linear) curves are shown on top of the raw data. **(E,F)** Phosphorylation of the Y292 and T300 residues has no effect on nuclear import. Representative images of S2R+ cells expressing wild type and mutant forms of moesin (green) under normal conditions and upon *Rae1* knockdown, and immunostained for GFP. The study of isoforms mutated to glutamic acid (Y292E and T300E) was carried out in a separate experiment. **(B,C,E,F)** Optical sections were obtained via confocal microscopy and one mid-plane is shown. Arrows point to nuclei. Scale bars: 25 μm. Graphs show the nuclear/cytoplasmic pixel intensity ratios. Data represent mean ± SD of three independent experiments (*n* = 3), derived from the analysis of 25 cells per sample (3 × 25 in total per condition). Kolmogorov-Smirnov test was used to test for normality of data distribution. Samples were compared pairwise by Student’s *t*-test [**(C,E)**: Moe, Y292A, Moe-T300D, **(F)**: Moe-Y292E, Moe-T300E] or Mann-Whitney U test [**(B,E)**: Moe-T300A, Moe-Y292D, **(F)**: Moe]. *p*-value: ***p < 0.001.

As has been established, as in the case of nucleoplasmin, that some proteins contain NLS motifs that are composed of two short amino acid sequences. The two parts are usually separated by a connecting region of 10–12 amino acids, so these localization sequences are called bipartite NLS (Lu et al., 2021). The linker of the bipartite NLS has traditionally been limited to 10–12 amino acids, but this length can vary (Lange et al., 2010; Yamano et al., 2020; Lu et al., 2021). In the case of moesin, there is a conserved KR motif 13 amino acids upstream from the NLS1 sequence (KR_X13_RRRK), at positions 279–280. The amino acid composition and position of the sequence meet the conditions established for bipartite NLS (Figure 2A). In addition, cNLS Mapper predicted this region as a bipartite NLS. To determine whether moesin’s NLS sequence is, in fact, bipartite, the KR_279-280_ residues were deleted and the nuclear accumulation of the resulting protein (Moe-DKR) was investigated by inducing nuclear import with *Rae1* RNAi (Figure 2B). The results showed that similarly to the Moe-DNLS form, Moe-DKR is not able to accumulate in the nucleus. This suggests that the KR and RRRK motifs together form the NLS1 sequence, so the NLS we have identified is bipartite. Pawlowski et al., have previously found that for actin-dependent nuclear imports of the MAL (also known as MRTF-A or MKL1) transcription cofactor, the bipartite NLS has a hierarchical structure, with the removal of each motif reducing MAL’s nuclear import to different degrees (Pawłowski et al., 2010). However, in the case of moesin, when RRRK and KR were removed, nearly identical fluorescence intensity ratios were measured in the nucleus (Figure 2B), indicating that the two parts of the NLS are interdependent and equally important for nuclear import. To further confirm that the KR_X13_RRRK_297_ motif is a functional NLS, a 27-amino acid fragment containing the moesin NLS was attached to the C-terminus of the green fluorescent (GFP) reporter protein, and the intracellular localization of the GFP-(Moe) NLS protein was monitored. Whereas the GFP protein itself is distributed in both the nucleus and cytoplasm of *Drosophila* S2R+ cells, with some accumulation in the nucleus (Figure 2C), GFP-(Moe)NLS was found to be highly concentrated in the nucleus (Figure 2C). This confirms that *Drosophila* moesin has a bipartite NLS, which is constructed of two clusters of basic amino acids, separated by a spacer of 13 amino acids.

When examining NLS motifs, deletion is a common method, however, to make it more clear that the deletions do not impede nuclear import because they disrupt the structure of the moesin protein, we analyzed the effect of deletions on the 3D structure using the AlphaFold2 software (Jumper et al., 2021; Varadi et al., 2022). We generated AlphaFold2 models using wild type (Supplementary Figure S1), DKR_279-280_, and DRRRK_294-297_ sequences of moesin. Each run resulted five models. Due to the deletion of KR amino acids (DKR), some side chain positions change (Supplementary Figure S3B), and the α1F3 alpha helix formed by the NLS becomes shorter, which results in a difference of 1 Å in the position of the backbone of the nearby β5F3 beta element (Supplementary Figure S3C). The relative position of the loop structure also shows more than 2 Å difference (Supplementary Figure S3C). This region is predicted with low confidence as it is part of the disordered region (Supplementary Figure S1) therefore, in this case the change might be due to the uncertainty in the prediction. The deletion of RRRK (DNLS) only changes the relative position of the coiled-coil (alpha-helix region) (Supplementary Figure S4). However, due to the disordered region, the exact position of this part of the molecule is unsure, therefore the real effect of this mutation on the position of the alpha-helix region remains uncertain. The simultaneous deletion of KR_279-280_, and RRRK_294-297_ residues does not generate additional changes in the structure (Supplementary Figure S5).

In sum, the structural analysis using AlphaFold2 predictions suggests that the deletions can cause structural changes, but they are unlikely to generate protein unfolding or damage moesin’s function. The deletion of KR_279-280_ has some effect on the structure but only in its vicinity and most of these changes are less than 2 Ångströms (Supplementary Figure S3C). The deletion of RRRK_294–297_ might affect the relative position of the coiled-coil alpha-helix region, but we have not enough information to conclude more. Considering that local structural changes smaller than 2-3 Ångströms might be significant in the case for instance of a catalytic pocket but can be tolerated by a flexible adaptor such as moesin, and 2.05 Å is the median resolution for X-ray crystallographic results in the Protein Data Bank, we can say that the deletions examined by us most likely have no serious effect on the structure of moesin.

The molecular weight of the untagged, endogenous moesin protein is ∼70 kDa, which is above the limit of passive diffusion. The GFP-tagged moesin used in our experiments has a molecular weight of ∼95 kDa; it certainly cannot diffuse freely into the nucleus. To confirm this with experimental data, we performed a fluorescence recovery after photobleaching (FRAP) experiment, in which the import dynamics of moesin and GFP, which enters the nucleus by passive diffusion, were compared. In these assays we irreversibly bleached the GFP signal in the entire nucleus of cultured *Drosophila* S2R+ cells with a few, high intensity laser pulses, and monitored the initial recovery of the fluorescent signal, which is the result of the import of unbleached molecules from the cytoplasm. The GFP protein recovered its nuclear fluorescence very rapidly, in about 2 min after bleaching, demonstrating constant and dynamic travelling (Figure 2D). However, we could observe only a low level of recovery of the moesin-GFP fluorescent signal, providing additional evidence that moesin is not passively diffusing into the nucleus. In fact, its continuous but moderate transport argues for cytoplasmic retention.

Phosphorylation of amino acids in the vicinity of NLS sequences may have a regulatory function, promoting or inhibiting the nuclear import of a given protein (Harreman et al., 2004; Nardozzi et al., 2010). In the case of moesin, a tyrosine (Y292) and a threonine (T300) are found near the NLS sequence (Figure 2A), therefore we investigated whether replacing them with aspartic acid (Y292D and T300D) or glutamic acid (Y292E and T300E), which mimic phosphorylation, or with non-phosphorylatable alanine (Y292A and T300A) affects the nuclear import of moesin. Analysis of the microscopic images showed that in all cases the amount of moesin in the nucleus increased significantly upon induction (Figures 2E, F) therefore, we concluded that the phosphorylation state of the tyrosine and threonine residues close to the NLS does not have a significant impact on nuclear import. These experiments also confirm that the effect on regulating the import previously observed for the KR_279-280_ motif is indeed specific and is not due to a change in the amino acid environment at the NLS region.

### 3.2 Regulation of moesin’s nuclear import

**FIGURE 3:**
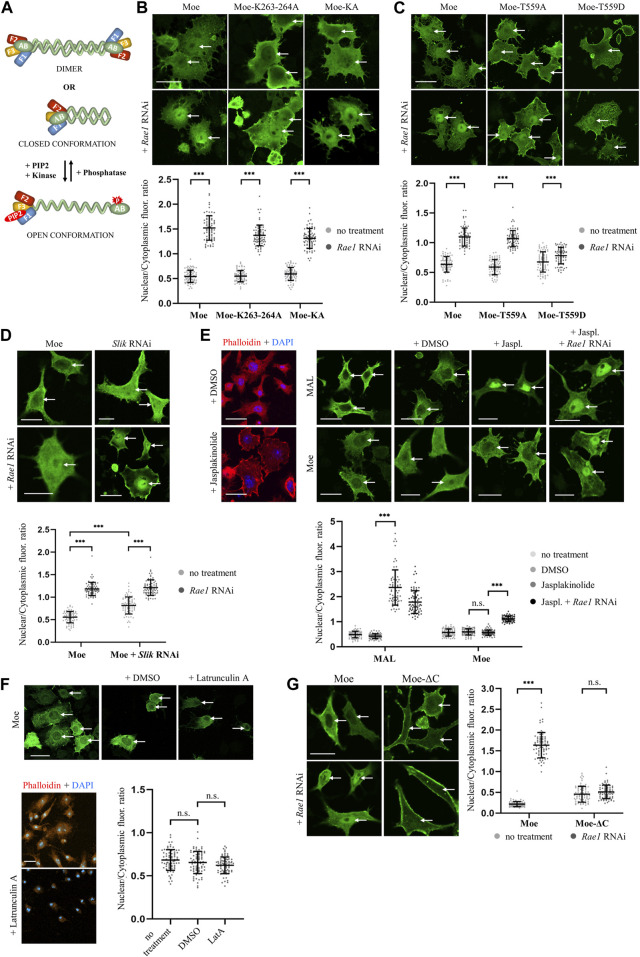
Moesin is imported into the nucleus in closed, monomeric form. **(A)** Conformational states of moesin. F1-F3—subdomains of the FERM domain, AB—Actin Binding domain, red star marks the phosphorylation at T559, red oval represents PIP2. **(B)** PIP2 binding is not necessary for nuclear entry. Representative images of S2R+ cells expressing wild type (Moe), Moe-K263-264A, and Moe-KA forms of moesin (green) under normal conditions and upon *Rae1* knockdown. **(C)** Phosphorylation at T559 hinders nuclear import. S2R+ cells expressing the wild type, and the T559A and T559D mutant forms of moesin (green). **(D)** The silencing of Slik kinase promotes the nuclear import of moesin. S2R+ cells expressing wild type moesin (green) without or together with *Slik* and *Rae1* RNAi. **(E)** Representative images of the experiment analyzing the import of moesin-GFP (green) under increased F-actin levels. Jasplakinolide treatment increases F-actin levels as revealed by phalloidin staining (red) and the nuclear accumulation of mouse MAL protein (positive control). DMSO: solvent of Jasplakinolide. Jaspl.—Jasplakinolide. **(F)** Representative images of the experiment analyzing the nuclear import of moesin-GFP (green) when the actin network is depolymerized with Latrunculin A treatment (controlled with phalloidin (red) staining). Decreased F-actin levels does not stimulate the nuclear import of moesin. DMSO—solvent of Latrunculin **(A)**. LatA—Latrunculin **(A)**. **(G)** S2R+ cells expressing GFP-tagged wild type (Moe) and C-terminally truncated (Moe-ΔC) moesin proteins (green). **(B–G)** Cells were immunostained with anti-GFP antibody. Optical sections were obtained via confocal microscopy and one mid-plane is shown. Nuclear import was induced by *Rae1* RNAi treatment. Arrows point to nuclei. Scale bars: 25 μm. Graphs show the quantification of immunostainings. Nuclear/cytoplasmic pixel intensity ratios were calculated. Data represent mean ± SD of three independent experiments (*n* = 3), derived from the analysis of 25 cells per sample (3 × 25 in total per condition). Kolmogorov-Smirnov test was used to test for normality of data distribution. Untreated and *Rae1* RNAi samples were compared pairwise by Student’s *t*-test (**(B)**, **(C)**: Moe-T559A, **(E)**: Moe + DMSO vs. Moe + Jasplakinolide, Moe + Jasplakinolide vs. Moe + *Rae1* RNAi; **(F)**, **(G)**: Moe-ΔC) or Mann-Whitney U test (**(C)**: Moe, Moe-T559D, **(E)**: MAL + DMSO vs. MAL + Jasplakinolide, **(G)**: Moe). In **(D)** two-way ANOVA analysis was performed by using the Aligned Rank Transformed (ART) N/CP ratio values. *p*-values: ***p < 0.001, and n.s. (not significant): *p* > 0.05.

ERM proteins are conformationally regulated. They exist in closed conformation (considered as inactive or dormant form) in which the C-terminal tail binds to and masks the N-terminal FERM domain (Figure 3A). Activation is mediated by the binding of phosphatidylinositol 4,5-bisphosphate (PIP2) and subsequent phosphorylation of a C-terminal threonine (T559 in *Drosophila* moesin), which stabilizes the open conformation (Ben-Aissa et al., 2012). The binding of PIP2 occurs in a sequential manner: PIP2 is transferred from the transient binding site (PATCH—lysines K254-255, K263-264) to the stable binding site (POCKET—lysines K61, K64, K279) (Ben-Aissa et al., 2012). Lysine in the KR_279-280_ motif, defined in previous experiments as part of the NLS, is a member of the POCKET binding site, which raises the possibility that the binding of PIP2 is necessary for the nuclear import of moesin. Therefore, we replaced one of the lysine doublets (Moe-K263-264A form), as well as both doublets at positions 254–255 and 263–264 (Moe-KA form) of the PATCH binding site, with alanine. Thus, by partially or fully abolishing the PATCH, we have created moesin forms in which the POCKET binding site is intact, but still not capable of PIP2 binding (Roch et al., 2010). The experiment with these mutant forms showed that, in response to *Rae1* RNAi treatment, Moe-K263-264A and MoeKA accumulate in the nucleus like the wild-type protein (Figure 3B). This confirms that the lack of KR_279-280_ amino acids inhibits nuclear import, not because of the lack of PIP2 binding, but because together with the RRRK_294–297_ amino acid cluster, KR_279-280_ forms a motif responsible for nuclear import, so the NLS of moesin is indeed bipartite.

The nuclear import of the MoeKA form also clarified that the binding of PIP2 is not required for nuclear import, which in turn suggests that the activation of moesin is not necessary for its nuclear entry. In order to test this idea, we generated protein variants in which the threonine responsible for activation was replaced by non-phosphorylatable alanine (MoeT559A—inactive form) or aspartic acid, which imitated a constant phosphorylation state (MoeT559D—constitutively active form) (Polesello et al., 2002). We then examined the nuclear import of these proteins by *Rae1* knock down. Wild-type and inactive MoeT559A forms were present in similar proportions in the nucleus, while MoeT559D showed much lower nuclear accumulation (Figure 3C), suggesting that moesin translocates into the nucleus in its inactive state. To confirm that closed conformation is preferred in nuclear import, we also examined how the inhibition of the Slik kinase, which phosphorylates T559 in *Drosophila* moesin and thereby stabilizes its opened state (Hipfner et al., 2004), affects the import. Simple main effects statistical analysis showed that both Rae1 and Slik RNAi treatment alone had a statistically significant effect on N/CP ratio (*p* = 2.2e-16 and 5.8e-15 respectively), which reveals that the knockdown of Slik alone increases the amount of moesin in the nucleus even without the induction of import. There was a statistically significant interaction also between the effects of Rae1 and Slik treatments (*p* = 1.2e-10), nuclear moesin levels were further increased by *Rae1* RNAi (Figure 3D). These results provide more evidence that the non-phosphorylated form of moesin is favored in nuclear import.

The weak nuclear import of the opened, activated moesin form can also be explained by the connection between the actin cytoskeleton and moesin, which might eclipse nuclear entry. Therefore, we investigated whether increasing the amount of F-actin in the cytoplasm, with the help of the actin filament stabilizing drug Jasplakinolide, has any effect on moesin’s nuclear import. To control the effect of the drug, the microfilament network was visualized with phalloidin staining (visualized by red color in Figure 3E), and nuclear localization of the mouse MAL protein was monitored (Miralles et al., 2003). As shown in Figure 3E, shifting the actin monomer/polymer balance towards F-actin with jasplakinolide induced strong nuclear accumulation of MAL, but appeared to have no effect on moesin’s nuclear import. Interestingly, depolymerization of the actin network by Latrunculin A treatment did not stimulate the nuclear import of moesin (Figure 3F). This suggests that it is not F-actin binding that inhibits the nuclear import of activated moesin. To further confirm this, we also examined the nuclear import of a truncated form of moesin (Moe-ΔC) that lacks the C-terminal F-actin binding domain and cannot acquire closed conformation. Moe-ΔC was unable to enter the nucleus upon induction of nuclear import (Figure 3G), providing further evidence that F-actin is not retaining moesin in the cytoplasm, and that closed conformation is favored for nuclear import.

The open conformation of ERM proteins allows for dimerization in a head-to-tail arrangement through the interaction between their N- and C-terminal domains, and some authors even suggest the possibility of oligomerization (Gary and Bretscher, 1995; Bhartur and Goldenring, 1998; Zhu et al., 2005). At the same time, ERMs are still able to interact with several of their binding partners after dimerization (Phang et al., 2016), so it can be assumed that some of its functions are also performed by *Drosophila* moesin in dimeric form. Although, our experiments showed that nuclear import occurs in closed conformation, and since dimerization requires open conformation, it is unlikely that moesin will enter the nucleus as a dimer. Our experiment analyzing predicted nuclear localization sequences was carried out in transfected cells expressing the wild-type, endogenous moesin protein in the background. Since Moe-DNLS was absent from the nucleus in these experiments, it is an obvious assumption that since the endogenous protein is not able to mediate the import of the NLS mutant protein, the monomeric form is preferred for nuclear entry. In line with this, the Moe-ΔC protein, which, due to the absence of its C-terminus, cannot form a closed conformation, nor dimerize, was unable to enter the nucleus. In summary, based on the findings that PIP2 binding and phosphorylation at T559, which are necessary for activation, are not needed for import, and that neither the dimer nor the truncated form capable of forming a closed conformation can be imported, we can conclude that the nuclear import of moesin takes place primarily in monomeric form, in its closed-conformational state.

### 3.3 The NLS, together with its surrounding region, is evolutionarily highly conserved

**FIGURE 4:**
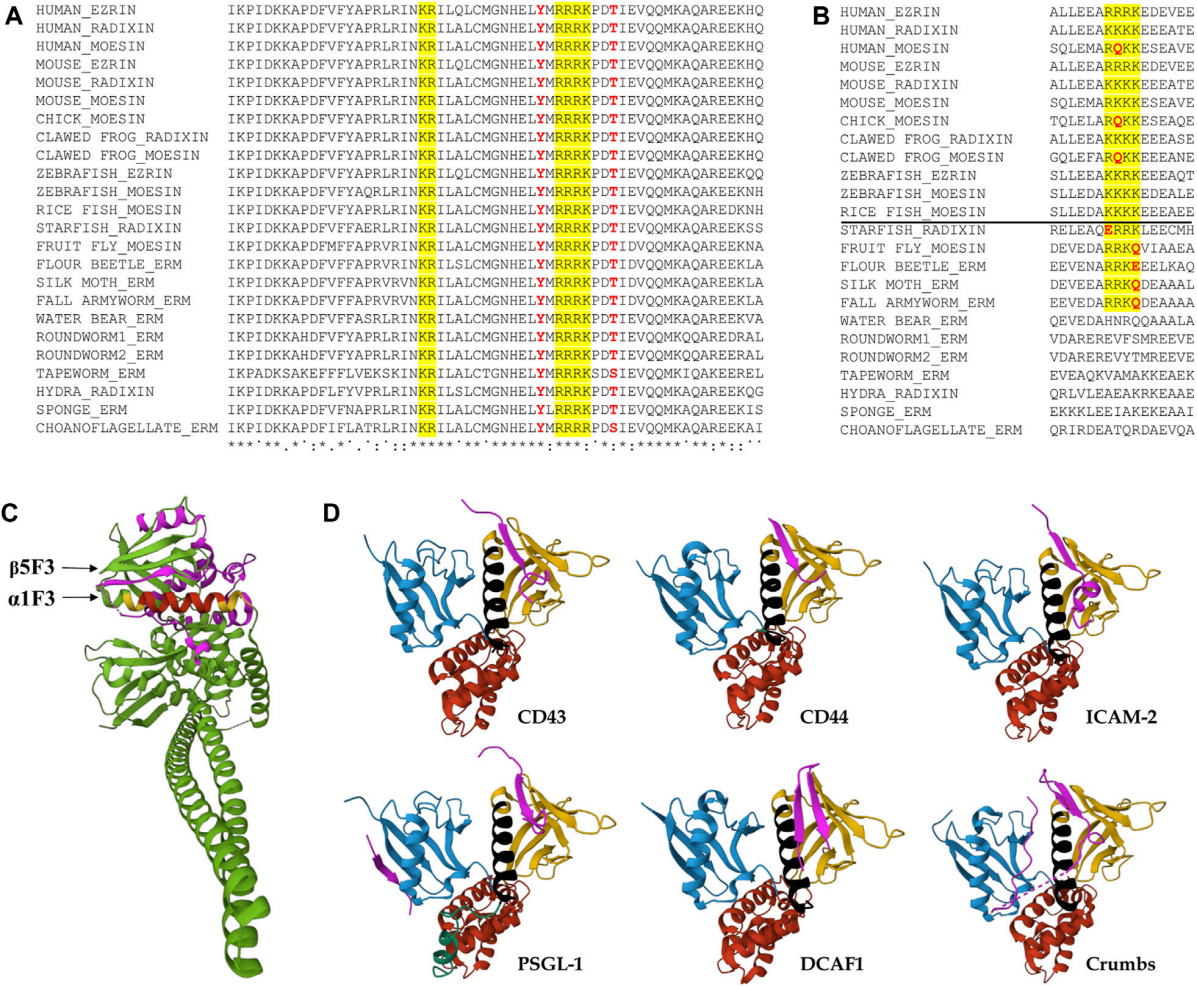
Evolutionary conservation of the region around the predicted NLS sequences, and the spatial position of the NLS. **(A)** Clustal Omega alignment of the protein sequences of the region surrounding the NLS reveals high evolutionary conservation from unicellular choanoflagellates through insects to humans. The bipartite NLS is highlighted in yellow, conserved phosphorylatable residues analyzed in this study are in red. Asterisks (*) indicate positions which have a single, fully conserved residue, colon (:) indicates conservation between groups of strongly similar properties, subscript period (.) indicates conservation between groups of weakly similar properties, superscript period (˙) indicates no conservation. Species names and protein accession numbers can be found in Table 1. **(B)** Multiple sequence alignment with Clustal Omega of the region containing the NLS2 sequence implicated in nuclear import in mammalian cells. The NLS is highlighted in yellow, non-conserved residues of the NLS are in red. The horizontal line separates vertebrate and invertebrate species. Species names and protein accession numbers can be found in Table 1. **(C)** The location of the NLS motif in the 3D protein structure. The structure was composed using data from the *Spodoptera frugiperda* full-length moesin (PDB accession 2I1K) (Li et al., 2007). The two parts of bipartite NLS are highlighted in yellow, the connecting region between them is in red, CTD is highlighted in magenta. **(D)** Representation of the FERM domain of ERMs or Merlin interacting with peptides (magenta) mimicking binding partner proteins CD43 (PDB accession 2EMS) (Takai et al., 2008), CD44 (PDB accession 2ZPY) (Mori et al., 2008), ICAM-2 (PDB accession IJ19) (Hamada et al., 2003), PSGL-1 (PDB accession 2EMT) (Takai et al., 2007), DCAF1 (PDB accession 4P7I) (Li et al., 2014), and Crumbs (PDB accession 4YL8) (Wei et al., 2015). FERM subdomains are colored as in Figures 3A, 5. The NLS is highlighted in black.

To see whether the bipartite NLS of *Drosophila* moesin exists in the ERM proteins of other species, thus to test the evolutionary conservation of the motif, we performed multiple sequence alignments of 24 ERM sequences from 18 different species. Comparison of the region surrounding the NLS revealed surprisingly high conservation in choanoflagellates, the closest living relatives of animals, and across metazoans (animals) from sponge to human ERMs (Figure 4A). We found that not only the two parts of the bipartite NLS motif, but the distance and the residues between them, as well as their immediate environment and the presence and position of the two phosphorylatable amino acids contained therein, are identical in all the proteins analyzed. In contrast to NLS1, the NLS2 motif, which was reported earlier as functional in human cells (Batchelor et al., 2004), is only weakly conserved (Figure 4B). Vertebrate ERM proteins from human, chicken, and clawed frog contain a glutamine in their NLS2 motif. In addition, the variability of the region corresponding to vertebrate NLS2 is very high in invertebrate ERMs. In fact, the NLS2 motif can only be recognized in star fish and insect proteins, but they also contain glutamine or glutamic acid residues within the sequence, raising doubts about the functionality of this motif (Figure 4B).

## 4 Discussion

Like their main binding partner, actin, ERM proteins also localize to the nucleus. Their concentration in the nucleus varies depending on the status of the cell, which suggests controlled nuclear transport (Batchelor et al., 2004; Kristó et al., 2017). In the work presented here, we aimed to explore the nuclear import mechanism of the only ERM protein of *Drosophila*. During the investigation of the nuclear import of *Drosophila* moesin, we tested three predicted NLS motifs and the N-terminus of the protein, then analyzed the exact sequence and regulation of the identified NLS, and finally, explored the effect of moesin’s activation on its nuclear import. Out of the four protein regions tested, only NLS1 (R_294_RRK_297_) was found to be a functional localization signal. Thirteen amino acid positions upstream from this sequence, we identified a second part (K_279_R_280_) of the motif, and confirmed that the NLS is in fact bipartite. We also showed that the potentially phosphorylatable tyrosine and threonine residues at positions 292 and 300, near the NLS, have no import regulatory function.

With the help of the constitutively activated MoeT559D and the non-phosphorylatable MoeT559A protein isoforms, we demonstrated that moesin translocates to the nucleus primarily in its closed, inactive form, and the activation of moesin inhibits import. This result is consistent with that described for Merlin/NF2, the closest relative of ERM proteins, which shows that the closed form of Merlin enters the nucleus (Li et al., 2010). The observations that the MoeKA isoform, which has no PIP2 binding capacity and is therefore certainly in closed conformation, can enter the nucleus just as well as the wild-type protein, that Moe-ΔC, which lacks the C-terminus necessary for acquiring the closed conformation, cannot enter the nucleus, and that the knock down of Slik kinase, responsible for the phosphorylation of moesin at T559, increases the amount of nuclear moesin, all provide further support for the view that the closed conformation is required for nuclear transport.

Our finding that NLS1 at amino acid positions 294–297 is functional, while NLS2 and NLS3 are not necessary for nuclear targeting, is in contrast to an earlier report by Batchelor and others (Batchelor et al., 2004). Multiple NLS sites were predicted in mammalian ERM proteins (Batchelor et al., 2004; Krawetz and Kelly, 2008), out of which the sequence corresponding to *Drosophila* NLS1, we report here, was shown to be non-functional, while the motif corresponding to *Drosophila* NLS2 was found necessary for nuclear localization (Batchelor et al., 2004). However, the nuclear localization of mammalian ERMs was studied only in cultured cells without the induction of nuclear import. On the other hand, the fact that the evolutionary conservation of NLS2 is restricted to vertebrates also supports that it can only be functional in vertebrates.

Although we managed to determine the NLS sequence in *Drosophila* moesin, our experiments showed that if the motif is deleted, albeit in small quantities, moesin is still present in the cell nucleus. The explanation for this is provided by earlier live imaging experiments which revealed that moesin is engulfed into the nucleus after mitosis, through associations with the chromosomes during the reorganization of the nucleus, and this process also occurs without the NLS sequence (Vilmos et al., 2009). In addition, it was also shown that most of the nuclear moesin proteins do not leave the nucleus (Kristó et al., 2017). Under normal conditions, this relatively low amount of nuclear moesin is sufficient to perform nuclear functions and the activation of nuclear import regulated by NLS is required only when transcriptional activity is greatly increased or nuclear mRNA export is impaired. Therefore, it is likely that in contrast to insects, mammalian ERMs require NLS2 for nuclear localization following cell division, while NLS1 is functional only under stress conditions. However, this is certainly not the case in *Drosophila*, as incorporation of moesin during anaphase occurs even in the absence of NLS2; Moe-DNLS2 is unambiguously present in the nucleus of interphase cells, as we demonstrated here (Figure 1B).

In the protein data bank, the 3D crystal structure is only available for one full-length ERM protein in its closed state, the moesin protein from the insect *S. frugiperda* (fall armyworm) (PDB accession 2I1K) (Li et al., 2007). Sequence conservation, biochemical results and structural analyses indicate that this structure represents a complete model for the closed and auto-inhibited conformation of all intact monomeric ERM proteins (Li et al., 2007; Michie et al., 2019). An investigation of the structure reveals that the bipartite NLS identified in our experiments is part of the FERM subdomain F3, and forms an alpha helix (α1F3) located on the surface of the FERM domain (Figure 4C). The accessibility of this region is slightly reduced in closed conformation due to the overlaying of the C-terminal domain (CTD) against the outer β sheet in the F3 subdomain (β5F3) (Li et al., 2007) (Figure 4C). Although our experiments do not provide direct evidence that nuclear import occurs only in closed conformation, they do indicate that closed conformation is preferred for nuclear entry, so it is also conceivable that the CTD close to the NLS stabilizes the interaction with the importin. Intriguingly, when we searched for conserved residues that could play such a stabilizing role, we found that the region of the CTD that lies around α1F3 in the closed state (V_467_-L_499_), both in terms of length and sequence, is the most variable part of the protein, and it does not show any signs of evolutionary conservation. In addition, all the software we used to analyze the sequence of both *Spodoptera* and *Drosophila* ERM proteins (IUPred3 (Erdos et al., 2021), PrDos (Ishida and Kinoshita, 2007), flDPnn (Hu et al., 2021), DisoMine (Orlando et al., 2022)) predict with high probability that this section is an intrinsically disordered region which extends between Q_450_-E_507_. Therefore, the spatial arrangement of the CTD around the NLS-containing α1F3 helix of *Spodoptera* might not reflect its exact position in other ERMs, including *Drosophila* moesin, and it most likely exhibits a flexible 3D structure. It is tempting to assume that the disordered region of the CTD that is close to α1F3 helps and, at the same time, might also regulate importin binding. However, the deletion of NLS3 (GDAGG_485-489_) from this region did not affect the nuclear import of moesin, which suggests that the CTD does not play a significant role in importin binding.

We found no evidence for the direct regulation of the NLS, and since our own unpublished observation shows that the import of moesin without induction is very weak, it can be assumed that something is retaining the protein in the cytoplasm. The weak nuclear import of the constitutively active MoeT559D form can be explained by the fact that the connection between the opened, activated moesin form and the actin cytoskeleton anchors moesin in the cytoplasm and thereby inhibits its nuclear transport. Although, this hypothesis is reinforced by work in which it was shown that MoeT559D binds to the F-actin network under the cell membrane in much larger quantities than the MoeT559A variant (Roch et al., 2010), our results that the depolymerization of actin has no effect on the nuclear import of moesin, and that the Moe-ΔC protein, which lacks the F-actin binding domain, is also unable to translocate into the nucleus contradicts this idea. In addition to F-actin, ERM proteins have numerous binding partners integrated in the cell membrane, to which they bind with their FERM domain (Michie et al., 2019). Thus, of course, it is also conceivable that the nuclear translocation of the activated moesin as well as the Moe-ΔC form is inhibited by these interactions. Binding of the FERM domain occurs in open state and requires PIP2 binding and phosphorylation of T559. Because nuclear import was observed with the isoforms which cannot bind PIP2 or cannot be phosphorylated, it is entirely reasonable to assume that retention by the interactions of the FERM domain, rather than the actin-binding domain, regulates the dynamics of moesin’s nuclear import. This idea is supported by the earlier finding that, in the case of ezrin, the turnover process due to association/dissociation with the membrane through the FERM domain is almost 10 times slower than the turnover of the interaction with the F-actin cortex (Fritzsche et al., 2014).

**FIGURE 5:**
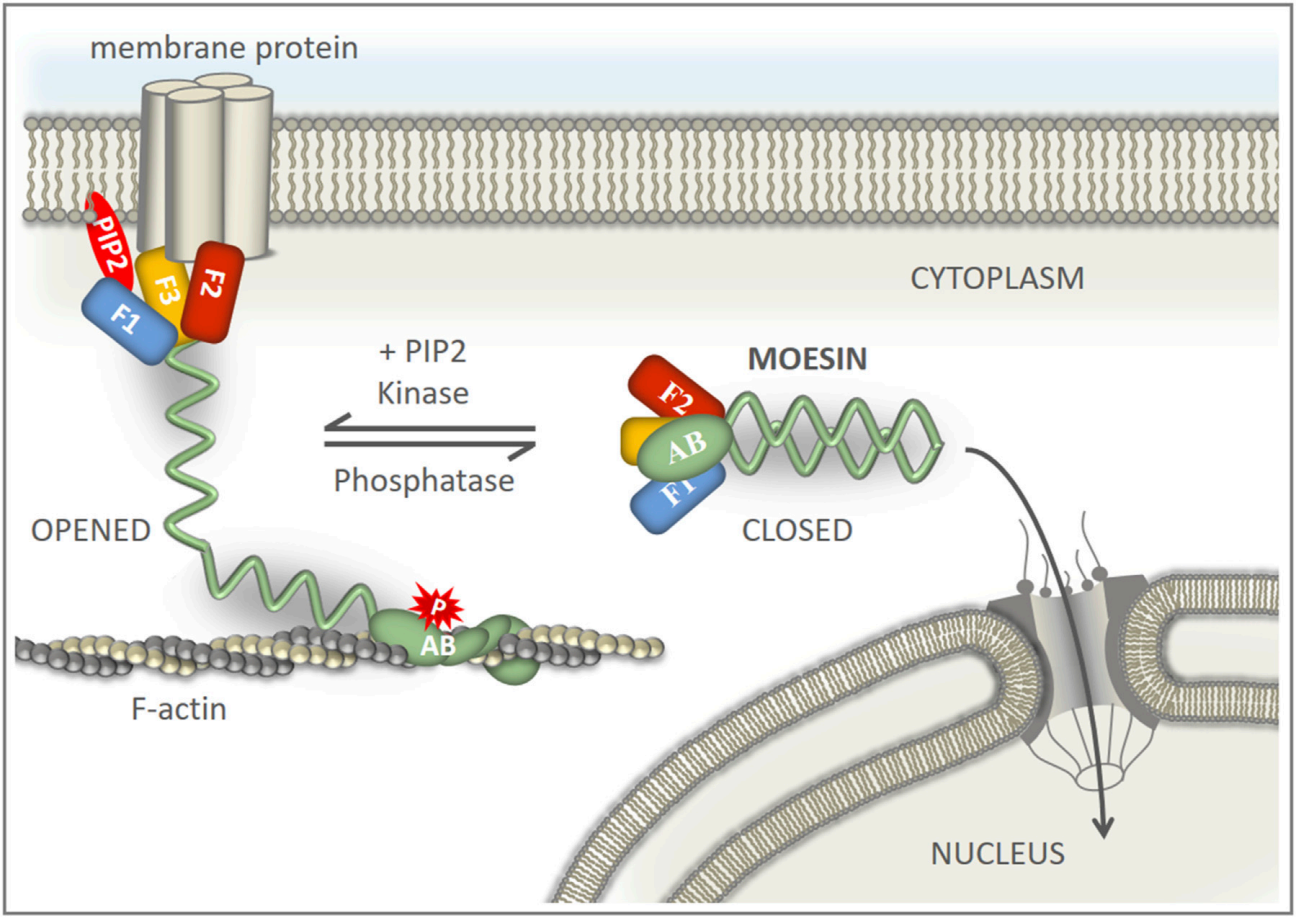
Model of the regulation of nuclear transport of moesin. PIP2 recruits moesin to the cell membrane from a cytoplasmic pool of dormant molecules. A stable association of phosphorylated moesin with the membrane and the actin cortex retains the protein in the cytoplasm. Dephosphorylation of moesin promotes closed conformation and detachment from the cell cortex. Closed moesin protein is mobile and can translocate into the nucleus.

Among the binding partners of the FERM domain of ERMs and Merlin (close relative of ERMs), CD43 (Takai et al., 2008), CD44 (Mori et al., 2008), ICAM-2 (Hamada et al., 2003), PSGL-1 (Takai et al., 2007), DDB1-and-Cullin-4-associated Factor 1 (DCAF1) (Li et al., 2014; Mori et al., 2014), and Crumbs (Wei et al., 2015) associate with the beta strand β5F3 in the groove which lies above the NLS-containing α1F3 alpha-helix of the FERM subdomain F3 (Figure 4D). Therefore, there is certainly competition between the importin and these binding partners. However, the conformational state of the protein is likely decisive between the two types of interactions. The open conformation is a prerequisite for the binding of membrane proteins, while the closed conformation is preferred for import as we demonstrated here. Therefore, we suggest that, ultimately, the balance between the phosphorylated and non-phosphorylated protein pools, set by the activity of specific kinases (Hipfner et al., 2004) and phosphatases (Kunda et al., 2012), determines the degree of nuclear import of moesin (Figure 5).

## Data Availability

The original contributions presented in the study are included in the article/Supplementary Material, further inquiries can be directed to the corresponding authors.

## References

[B1] Bajusz C. Kristó I. Abonyi C. Venit T. Vedelek V. Lukácsovich T. (2021). The nuclear activity of the actin-binding Moesin protein is necessary for gene expression in Drosophila. FEBS J. 288, 4812–4832. 10.1111/febs.15779 33606336

[B2] Batchelor C. L. Woodward A. M. Crouch D. H. (2004). Nuclear ERM (ezrin, radixin, moesin) proteins: regulation by cell density and nuclear import. Exp. Cell Res. 296, 208–222. 10.1016/j.yexcr.2004.02.010 15149851

[B3] Ben-Aissa K. Patino-Lopez G. Belkina N. V. Maniti O. Rosales T. Hao J. J. (2012). Activation of moesin, a protein that links actin cytoskeleton to the plasma membrane, occurs by phosphatidylinositol 4,5-bisphosphate (PIP2) binding sequentially to two sites and releasing an autoinhibitory linker. J. Biol. Chem. 287, 16311–16323. 10.1074/JBC.M111.304881 22433855 PMC3351316

[B4] Bergquist J. Gobom J. Blomberg A. Roepstorff P. Ekman R. (2001). Identification of nuclei associated proteins by 2D-gel electrophoresis and mass spectrometry. J. Neurosci. Methods 109, 3–11. 10.1016/S0165-0270(01)00395-8 11489294

[B5] Berman H. M. Westbrook J. Feng Z. Gilliland G. Bhat T. N. Weissig H. (2000). The protein Data Bank. Nucleic Acids Res. 28, 235–242. 10.1093/NAR/28.1.235 10592235 PMC102472

[B6] Bhartur S. G. Goldenring J. R. (1998). Mapping of ezrin dimerization using yeast two-hybrid screening. Biochem. Biophys. Res. Commun. 243, 874–877. 10.1006/BBRC.1998.8196 9501018

[B7] Bohnsack M. T. Stüven T. Kuhn C. Cordes V. C. Görlich D. (2006). A selective block of nuclear actin export stabilizes the giant nuclei of Xenopus oocytes. Nat. Cell Biol. 8, 257–263. 10.1038/NCB1357 16489345

[B8] Bosanquet D. C. Ye L. Harding K. G. Jiang W. G. (2014). FERM family proteins and their importance in cellular movements and wound healing (review). Int. J. Mol. Med. 34, 3–12. 10.3892/IJMM.2014.1775 24820650

[B9] Brameier M. Krings A. MacCallum R. M. (2007). NucPred—predicting nuclear localization of proteins. Bioinformatics 23, 1159–1160. 10.1093/BIOINFORMATICS/BTM066 17332022

[B73] Collier S. Chan H. Y. Toda T. McKimmie C. Johnson G. Adler P. N. (2012). The Drosophila embargoed gene is required for larval progression and encodes the functional homolog of schizosaccharomyces Crm1. Genetics. 155 (4), 1799–807. 10.1093/genetics/155.4.1799 PMC1461193 10924475

[B10] Day C. A. Kraft L. J. Kang M. Kenworthy A. K. (2012). Analysis of protein and lipid dynamics using confocal fluorescence recovery after photobleaching (FRAP). Curr. Protoc. Cytom. Chapter 2, Unit 2.19. 10.1002/0471142956.cy0219s62 PMC3538152 23042527

[B11] Di Cristofano C. Leopizzi M. Miraglia A. Sardella B. Moretti V. Ferrara A. (2010). Phosphorylated ezrin is located in the nucleus of the osteosarcoma cell. Mod. Pathol. 23, 1012–1020. 10.1038/modpathol.2010.77 20348881

[B12] Dopie J. Skarp K. P. Rajakylä E. K. Tanhuanpää K. Vartiainen M. K. (2012). Active maintenance of nuclear actin by importin 9 supports transcription. Proc. Natl. Acad. Sci. U. S. A. 109, E544–E552. 10.1073/pnas.1118880109 22323606 PMC3295300

[B13] Erdos G. Pajkos M. Dosztányi Z. (2021). IUPred3: prediction of protein disorder enhanced with unambiguous experimental annotation and visualization of evolutionary conservation. Nucleic Acids Res. 49, W297–W303. 10.1093/NAR/GKAB408 34048569 PMC8262696

[B15] Fritzsche M. Thorogate R. Charras G. (2014). Quantitative analysis of ezrin turnover dynamics in the actin cortex. Biophys. J. 106, 343–353. 10.1016/J.BPJ.2013.11.4499 24461009 PMC3907236

[B16] García-Ortiz A. Serrador J. M. (2020). ERM proteins at the crossroad of leukocyte polarization, migration and intercellular adhesion. Int. J. Mol. Sci. 21, 1502. 10.3390/IJMS21041502 32098334 PMC7073024

[B17] Gary R. Bretscher A. (1995). Ezrin self-association involves binding of an N-terminal domain to a normally masked C-terminal domain that includes the F-actin binding site. Mol. Biol. Cell 6, 1061–1075. 10.1091/MBC.6.8.1061 7579708 PMC301263

[B18] Gunasekaran S. Miyagawa Y. Miyamoto K. (2022). Actin nucleoskeleton in embryonic development and cellular differentiation. Curr. Opin. Cell Biol. 76, 102100. 10.1016/j.ceb.2022.102100 35605340

[B19] Hamada K. Shimizu T. Yonemura S. Tsukita S. Tsukita S. Hakoshima T. (2003). Structural basis of adhesion-molecule recognition by ERM proteins revealed by the crystal structure of the radixin–ICAM-2 complex. EMBO J. 22, 502–514. 10.1093/EMBOJ/CDG039 12554651 PMC140724

[B20] Harreman M. T. Kline T. M. Milford H. G. Harben M. B. Hodel A. E. Corbett A. H. (2004). Regulation of nuclear import by phosphorylation adjacent to nuclear localization signals. J. Biol. Chem. 279, 20613–20621. 10.1074/JBC.M401720200 14998990

[B21] Hipfner D. R. Keller N. Cohen S. M. (2004). Slik Sterile-20 kinase regulates Moesin activity to promote epithelial integrity during tissue growth. Genes Dev. 18, 2243–2248. 10.1101/GAD.303304 15371338 PMC517517

[B72] Hofmann W. A. Arduini A. Nicol S. M. Camacho C. J. Lessard J. L. Fuller-Pace F. V. (2009). SUMOylation of nuclear actin. J. Cell Biol. 186 (2), 193–200. 10.1083/jcb.200905016 19635839 PMC2717643

[B22] Houtsmuller A. B. Vermeulen W. (2001). Macromolecular dynamics in living cell nuclei revealed by fluorescence redistribution after photobleaching. Histochem Cell Biol. 115 (1), 13–21. 10.1007/s004180000234 11219603

[B23] Hu G. Katuwawala A. Wang K. Wu Z. Ghadermarzi S. Gao J. (2021). flDPnn: accurate intrinsic disorder prediction with putative propensities of disorder functions. Nat. Commun. 121 (12), 4438–8. 10.1038/s41467-021-24773-7 PMC8295265 34290238

[B25] Ishida T. Kinoshita K. (2007). PrDOS: prediction of disordered protein regions from amino acid sequence. Nucleic Acids Res. 35, W460–W464. 10.1093/NAR/GKM363 17567614 PMC1933209

[B26] Ivetic A. Ridley A. J. (2004). Ezrin/radixin/moesin proteins and Rho GTPase signalling in leucocytes. Immunology 112, 165–176. 10.1111/J.1365-2567.2004.01882.X 15147559 PMC1782489

[B27] Jankovics F. Sinka R. Lukácsovich T. Erdélyi M. (2002). MOESIN crosslinks actin and cell membrane in Drosophila oocytes and is required for OSKAR anchoring. Curr. Biol. 12, 2060–2065. 10.1016/S0960-9822(02)01256-3 12477397

[B28] Jumper J. Evans R. Pritzel A. Green T. Figurnov M. Ronneberger O. (2021). Highly accurate protein structure prediction with AlphaFold. Nature 596 (7873), 583–589. 10.1038/s41586-021-03819-2 34265844 PMC8371605

[B29] Kaul S. C. Kawai R. Nomura H. Mitsui Y. Reddel R. R. Wadhwa R. (1999). Identification of a 55-kDa ezrin-related protein that induces cytoskeletal changes and localizes to the nucleolus. Exp. Cell Res. 250, 51–61. 10.1006/EXCR.1999.4491 10388520

[B30] Kosugi S. Hasebe M. Tomita M. Yanagawa H. (2009). Systematic identification of cell cycle-dependent yeast nucleocytoplasmic shuttling proteins by prediction of composite motifs. Proc. Natl. Acad. Sci. U. S. A. 106, 10171–10176. 10.1073/PNAS.0900604106 19520826 PMC2695404

[B31] Krawetz R. Kelly G. M. (2008). Moesin signalling induces F9 teratocarcinoma cells to differentiate into primitive extraembryonic endoderm. Cell. Signal. 20, 163–175. 10.1016/J.CELLSIG.2007.10.011 17993262

[B32] Kristó I. Bajusz C. Borsos B. N. Pankotai T. Dopie J. Jankovics F. (2017). The actin binding cytoskeletal protein Moesin is involved in nuclear mRNA export. Biochim. Biophys. acta. Mol. Cell Res. 1864, 1589–1604. 10.1016/J.BBAMCR.2017.05.020 28554770

[B33] Kristó I. Bajusz I. Bajusz C. Borkúti P. Vilmos P. (2016). Actin, actin-binding proteins, and actin-related proteins in the nucleus. Histochem. Cell Biol. 145, 373–388. 10.1007/S00418-015-1400-9 26847179

[B74] Kunda P. Rodrigues N. T. Moeendarbary E. Liu T. Ivetic A. Charras G. (2012). PP1-mediated moesin dephosphorylation couples polar relaxation to mitotic exit. Curr. Biol. 22 (3), 231–6. 10.1016/j.cub.2011.12.016 22209527

[B34] Kumeta M. Yoshimura S. H. Hejna J. Takeyasu K. (2012). Nucleocytoplasmic shuttling of cytoskeletal proteins: molecular mechanism and biological significance. Int. J. Cell Biol. 2012, 494902. 10.1155/2012/494902 22229032 PMC3249633

[B35] Lange A. McLane L. M. Mills R. E. Devine S. E. Corbett A. H. (2010). Expanding the definition of the classical bipartite nuclear localization signal. Traffic 11, 311–323. 10.1111/J.1600-0854.2009.01028.X 20028483 PMC2886731

[B36] Li Q. Nance M. R. Kulikauskas R. Nyberg K. Fehon R. Karplus P. A. (2007). Self-masking in an intact ERM-merlin protein: an active role for the central alpha-helical domain. J. Mol. Biol. 365, 1446–1459. 10.1016/J.JMB.2006.10.075 17134719 PMC1796844

[B37] Li W. Cooper J. Karajannis M. A. Giancotti F. G. (2012). Merlin: a tumour suppressor with functions at the cell cortex and in the nucleus. EMBO Rep. 13, 204–215. 10.1038/EMBOR.2012.11 22482125 PMC3323126

[B38] Li W. You L. Cooper J. Schiavon G. Pepe-Caprio A. Zhou L. (2010). Merlin/NF2 suppresses tumorigenesis by inhibiting the E3 ubiquitin ligase CRL4(DCAF1) in the nucleus. Cell 140 (4), 477–490. 10.1016/j.cell.2010.01.029 20178741 PMC2828953

[B39] Li Y. Wei Z. Zhang J. Yang Z. Zhang M. (2014). Structural basis of the binding of merlin FERM domain to the E3 ubiquitin ligase substrate adaptor DCAF1. J. Biol. Chem. 289, 14674–14681. 10.1074/JBC.M114.551184 24706749 PMC4031523

[B40] Lu J. Wu T. Zhang B. Liu S. Song W. Qiao J. (2021). Types of nuclear localization signals and mechanisms of protein import into the nucleus. Cell Commun. Signal. 19, 60–10. 10.1186/S12964-021-00741-Y 34022911 PMC8140498

[B41] Madeira F. Pearce M. Tivey A. R. N. Basutkar P. Lee J. Edbali O. (2022). Search and sequence analysis tools services from EMBL-EBI in 2022. Nucleic Acids Res. 50, W276–W279. 10.1093/NAR/GKAC240 35412617 PMC9252731

[B42] Melendez-Vasquez C. V. Rios J. C. Zanazzi G. Lambert S. Bretscher A. Salzer J. L. (2001). Nodes of Ranvier form in association with ezrin-radixin-moesin (ERM)-positive Schwann cell processes. Proc. Natl. Acad. Sci. U. S. A. 98, 1235–1240. 10.1073/PNAS.98.3.1235 11158623 PMC14738

[B43] Michie K. A. Bermeister A. Robertson N. O. Goodchild S. C. Curmi P. M. G. (2019). Two sides of the coin: ezrin/radixin/moesin and merlin control membrane structure and contact inhibition. Int. J. Mol. Sci. 20, 1996. 10.3390/IJMS20081996 31018575 PMC6515277

[B44] Miralles F. Posern G. Zaromytidou A. I. Treisman R. (2003). Actin dynamics control SRF activity by regulation of its coactivator MAL. Cell 113, 329–342. 10.1016/S0092-8674(03)00278-2 12732141

[B45] Mori T. Gotoh S. Shirakawa M. Hakoshima T. (2014). Structural basis of DDB1-and-Cullin 4-associated Factor 1 (DCAF1) recognition by merlin/NF2 and its implication in tumorigenesis by CD44-mediated inhibition of merlin suppression of DCAF1 function. Genes Cells 19, 603–619. 10.1111/GTC.12161 24912773

[B46] Mori T. Kitano K. Terawaki S. I. Maesaki R. Fukami Y. Hakoshima T. (2008). Structural basis for CD44 recognition by ERM proteins. J. Biol. Chem. 283, 29602–29612. 10.1074/JBC.M803606200 18753140 PMC2662033

[B47] Nardozzi J. D. Lott K. Cingolani G. (2010). Phosphorylation meets nuclear import: a review. Cell Commun. Signal. 8, 32–17. 10.1186/1478-811X-8-32 21182795 PMC3022542

[B48] Orlando G. Raimondi D. Codicè F. Tabaro F. Vranken W. (2022). Prediction of disordered regions in proteins with recurrent neural networks and protein dynamics. J. Mol. Biol. 434, 167579. 10.1016/J.JMB.2022.167579 35469832

[B49] Pawłowski R. Rajakylä E. K. Vartiainen M. K. Treisman R. (2010). An actin-regulated importin α/β-dependent extended bipartite NLS directs nuclear import of MRTF-A. EMBO J. 29, 3448–3458. 10.1038/EMBOJ.2010.216 20818336 PMC2964165

[B50] Percipalle P. Vartiainen M. (2019). Cytoskeletal proteins in the cell nucleus: a special nuclear actin perspective. Mol. Biol. Cell 30, 1781–1785. 10.1091/mbc.E18-10-0645 31306096 PMC6727747

[B51] Phang J. M. Harrop S. J. Duff A. P. Sokolova A. V. Crossett B. Walsh J. C. (2016). Structural characterization suggests models for monomeric and dimeric forms of full-length ezrin. Biochem. J. 473, 2763–2782. 10.1042/BCJ20160541 27364155

[B52] Polesello C. Delon I. Valenti P. Ferrer P. Payre F. (2002). Dmoesin controls actin-based cell shape and polarity during *Drosophila melanogaster* oogenesis. Nat. Cell Biol. 4, 782–789. 10.1038/NCB856 12360288

[B54] Roch F. Polesello C. Roubinet C. Martin M. Roy C. Valenti P. (2010). Differential roles of PtdIns(4,5)P2 and phosphorylation in moesin activation during Drosophila development. J. Cell Sci. 123, 2058–2067. 10.1242/JCS.064550 20519583

[B56] Schneider C. A. Rasband W. S. Eliceiri K. W. (2012). NIH Image to ImageJ: 25 years of image analysis. Nat. Methods 9 (7), 671–675. 10.1038/nmeth.2089 22930834 PMC5554542

[B57] Sehnal D. Bittrich S. Deshpande M. Svobodová R. Berka K. Bazgier V. (2021). Mol* Viewer: modern web app for 3D visualization and analysis of large biomolecular structures. Nucleic Acids Res. 49, W431–W437. 10.1093/NAR/GKAB314 33956157 PMC8262734

[B58] Shabardina V. Kashima Y. Suzuki Y. Makalowski W. (2020). Emergence and evolution of ERM proteins and merlin in metazoans. Genome Biol. Evol. 12, 3710–3724. 10.1093/GBE/EVZ265 31851361 PMC6978628

[B59] Song Y. Ma X. Zhang M. Wang M. Wang G. Ye Y. (2020). Ezrin mediates invasion and metastasis in tumorigenesis: a review. Front. Cell Dev. Biol. 8, 588801. 10.3389/fcell.2020.588801 33240887 PMC7683424

[B60] Stüven T. Hartmann E. Görlich D. (2003). Exportin 6: a novel nuclear export receptor that is specific for profilin.actin complexes. EMBO J. 22, 5928–5940. 10.1093/EMBOJ/CDG565 14592989 PMC275422

[B61] Takai Y. Kitano K. Terawaki S. Maesaki R. Hakoshima T. (2008). Structural basis of the cytoplasmic tail of adhesion molecule CD43 and its binding to ERM proteins. J. Mol. Biol. 381, 634–644. 10.1016/J.JMB.2008.05.085 18614175

[B62] Takai Y. Kitano K. Terawaki S. I. Maesaki R. Hakoshima T. (2007). Structural basis of PSGL-1 binding to ERM proteins. Genes Cells 12, 1329–1338. 10.1111/J.1365-2443.2007.01137.X 18076570

[B63] UniProt Consortium (2023). UniProt: the universal protein knowledgebase in 2023. Nucleic Acids Res. 51, D523–D531. 10.1093/NAR/GKAC1052 36408920 PMC9825514

[B64] Varadi M. Anyango S. Deshpande M. Nair S. Natassia C. Yordanova G. (2022). AlphaFold Protein Structure Database: massively expanding the structural coverage of protein-sequence space with high-accuracy models. Nucleic Acids Res. 50 (D1), D439–D444. 10.1093/nar/gkab1061 34791371 PMC8728224

[B65] Vilmos P. Jankovics F. Szathmári M. Lukácsovich T. Henn L. Erdélyi M. (2009). Live imaging reveals that the Drosophila actin-binding ERM protein, moesin, co-localizes with the mitotic spindle. Eur. J. Cell Biol. 88, 609–619. 10.1016/J.EJCB.2009.05.006 19592131

[B66] Vilmos P. Kristó I. Szikora S. Jankovics F. Lukácsovich T. Kari B. (2016). The actin-binding ERM protein Moesin directly regulates spindle assembly and function during mitosis. Cell Biol. Int. 40, 696–707. 10.1002/CBIN.10607 27006187

[B67] Wada A. Fukuda M. Mishima M. Nishida E. (1998). Nuclear export of actin: a novel mechanism regulating the subcellular localization of a major cytoskeletal protein. EMBO J. 17, 1635–1641. 10.1093/emboj/17.6.1635 9501085 PMC1170511

[B68] Wei Z. Li Y. Ye F. Zhang M. (2015). Structural basis for the phosphorylation-regulated interaction between the cytoplasmic tail of cell polarity protein Crumbs and the actin-binding protein moesin. J. Biol. Chem. 290, 11384–11392. 10.1074/JBC.M115.643791 25792740 PMC4416843

[B69] Wobbrock J. O. Findlater L. Gergle D. Higgins J. J. (2011). “The aligned rank transform for nonparametric factorial analyses using only ANOVA procedures,” in Proceedings of the ACM Conference on Human Factors in Computing Systems (CHI '11), Vancouver, British Columbia, May 7-12, 2011 (New York: ACM Press), 143–146.

[B70] Yamano S. Kimura M. Chen Y. Imamoto N. Ohki R. (2020). Nuclear import of IER5 is mediated by a classical bipartite nuclear localization signal and is required for HSF1 full activation. Exp. Cell Res. 386, 111686. 10.1016/J.YEXCR.2019.111686 31669744

[B71] Zhu L. Liu Y. Forte J. G. (2005). Ezrin oligomers are the membrane-bound dormant form in gastric parietal cells. Am. J. Physiol. - Cell Physiol. 288, 1242–1254. 10.1152/AJPCELL.00521.2004 15788482

