# Supplementary material for: A bipartite NLS motif mediates the nuclear import of Drosophila moesin
Source: Front Cell Dev Biol. 2024 Feb 21;12:1206067. doi: 10.3389/fcell.2024.1206067 (PMC10915024; doi:10.3389/fcell.2024.1206067)
Supplement: Supplementary file 1 [file Table1.DOCX]

Supplementary Material

A bipartite NLS motif mediates the nuclear import of Drosophila moesin

Zoltán Kovács, Csaba Bajusz, Anikó Szabó, Péter Borkúti, Balázs Vedelek, Réka Benke, Zoltán Lipinszki, Ildikó Kristó*, Péter Vilmos*

*** Correspondence:** Péter Vilmos vilmosp@brc.hu, Ildikó Kristó kristo.ildiko@brc.hu


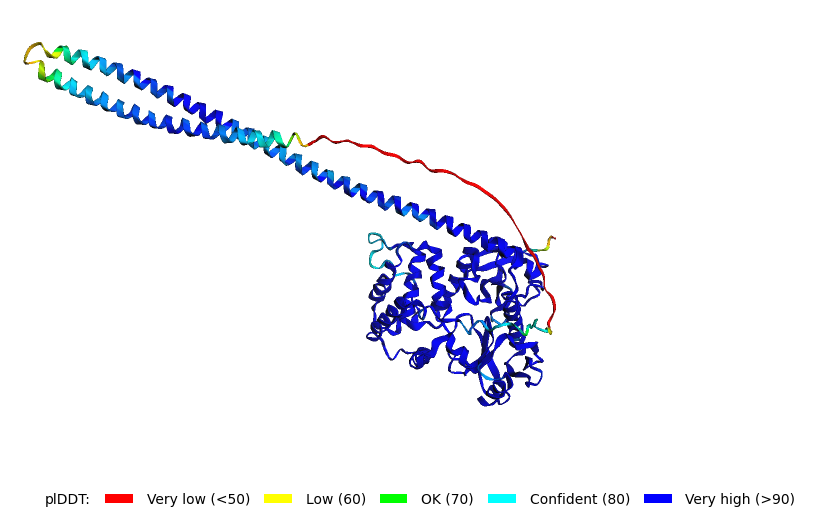


**B.**

**A.**


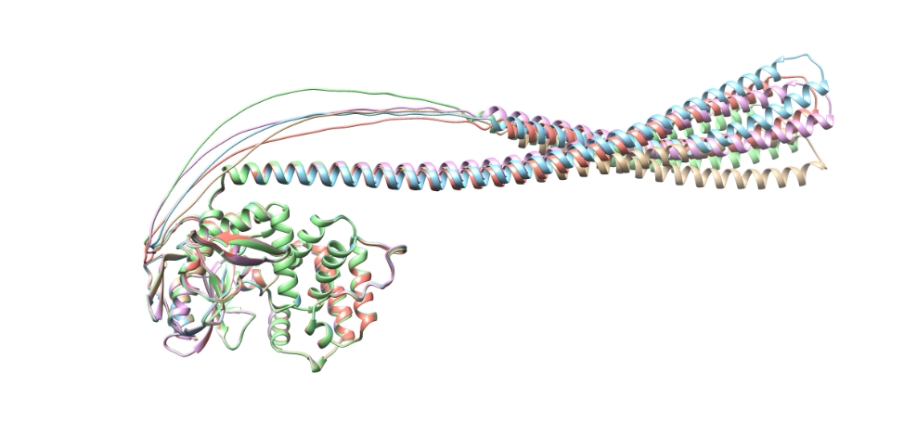


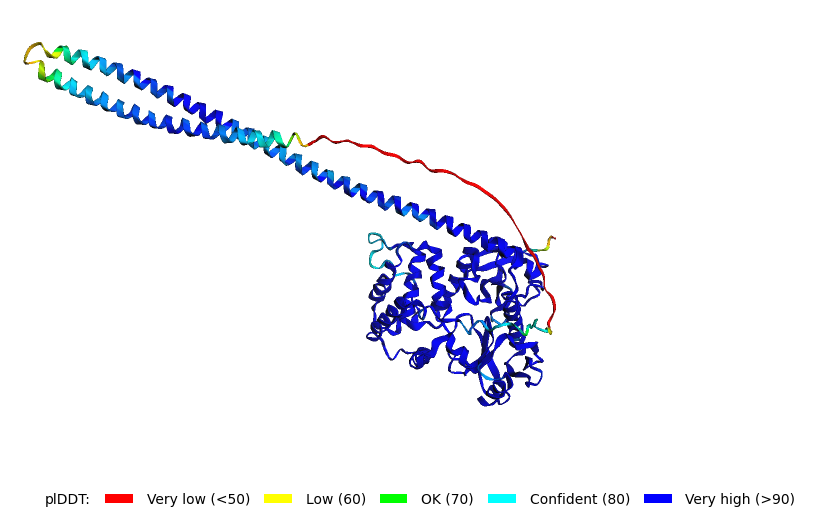

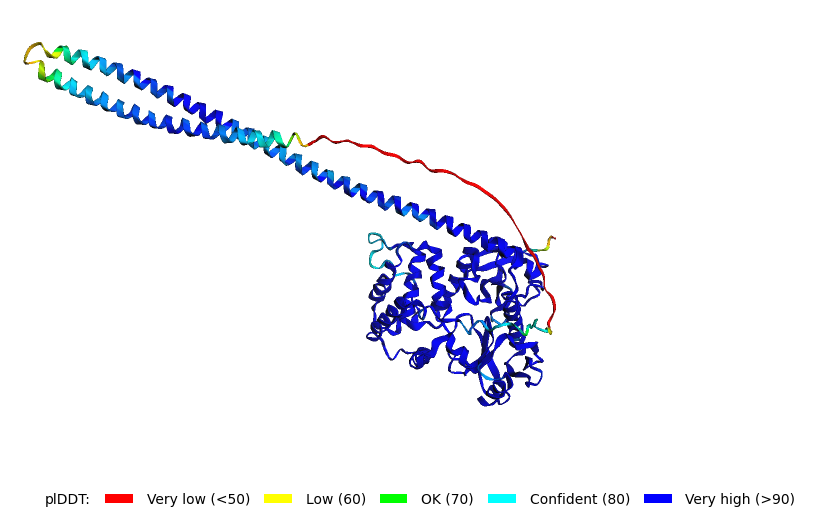


**Figure S1. AlphaFold2 models of full-length, wild type (wt) Drosophila moesin protein.** **(A)** Top five AlphaFold2 generated moesin models. **(B)** Top moesin AlphaFold2 model coloured by confidency.

**(Å)**


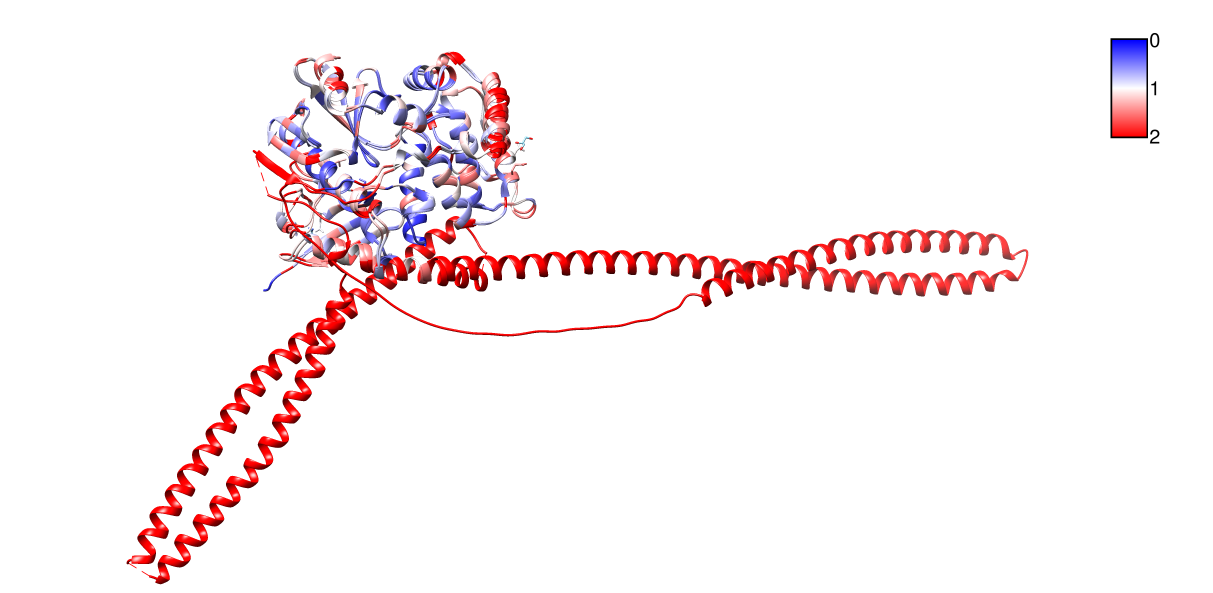

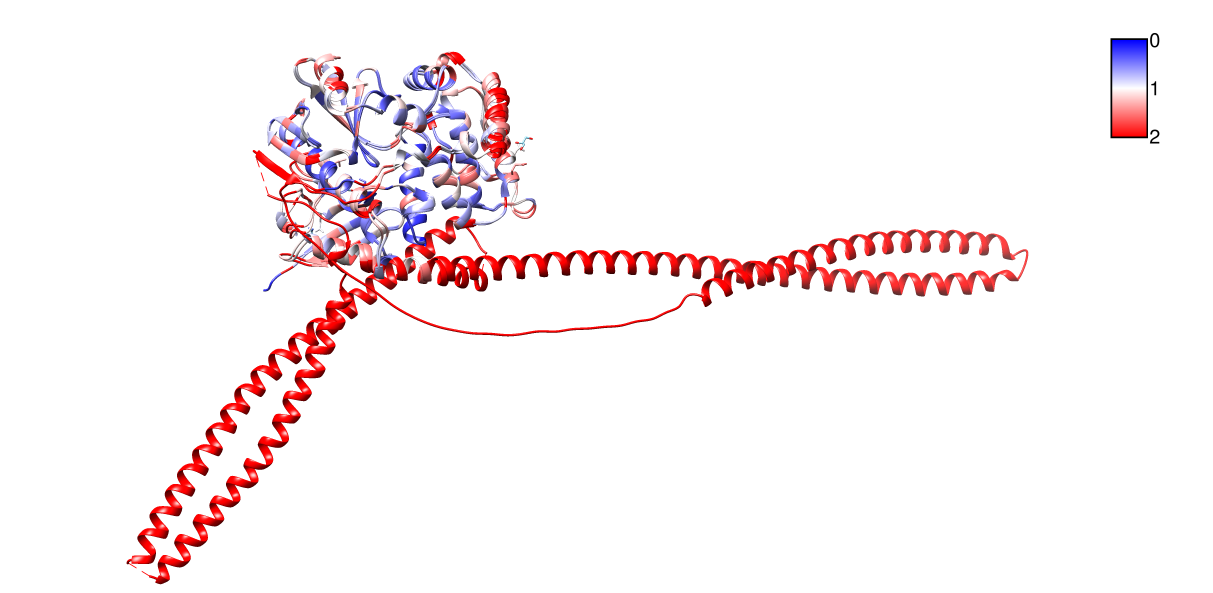


**Figure S2. Overlay of 2I1K structure of the RCSB Protein Data Bank (PDB) and wt Drosophila AlphaFold2 models.** The models are coloured according to their spatial difference Root Mean Square Deviation (RMSD) calculation, scale bar refers to Ångström.


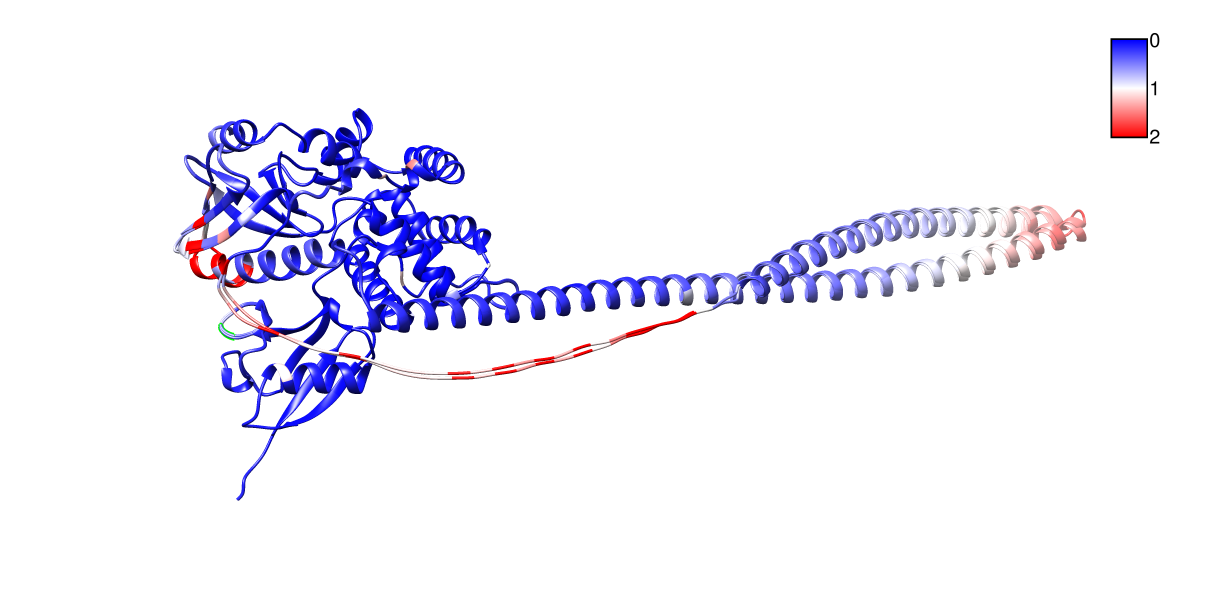


**B.**

**(Å)**

**A.**

**C.**

**(Å)**

**(Å)**


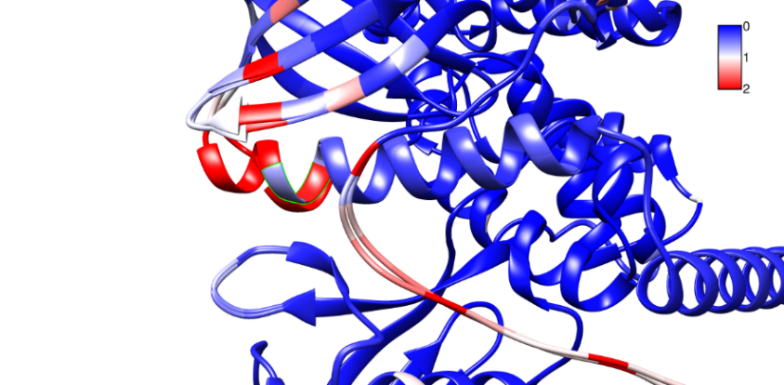


β5F3

α1F3

β5F3

α1F3


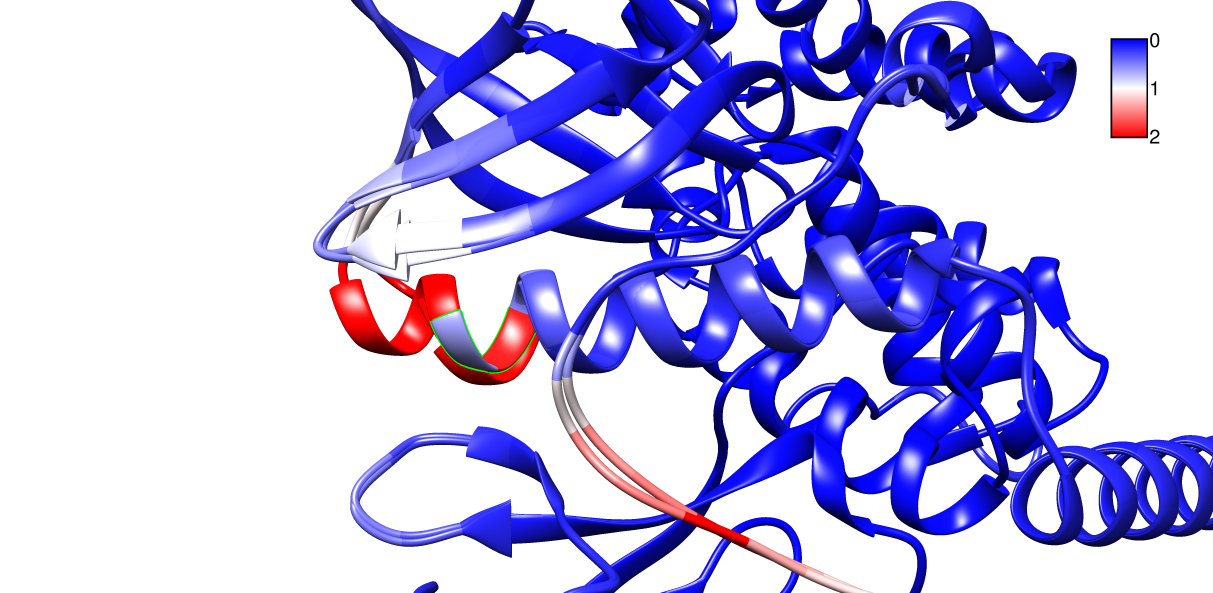


**Figure S3. Deletion of the KR_279-280_ amino acids of the NLS (Moe-DKR) cause only minor structural changes locally.** **(A-B)** shows RMSD calculated for all atoms while in C) RMSD for the backbone is shown. Scale bars refer to Ångström.


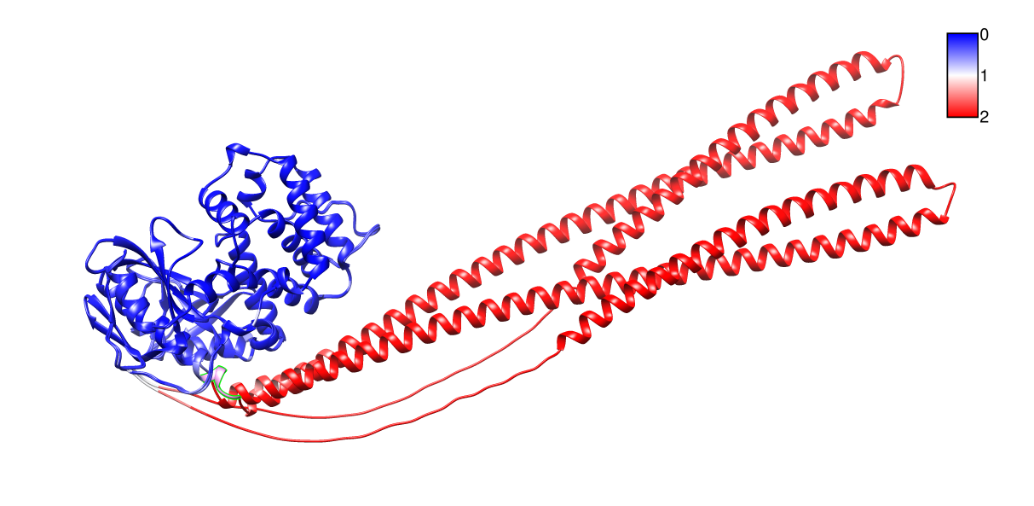


**A.**

**B.**


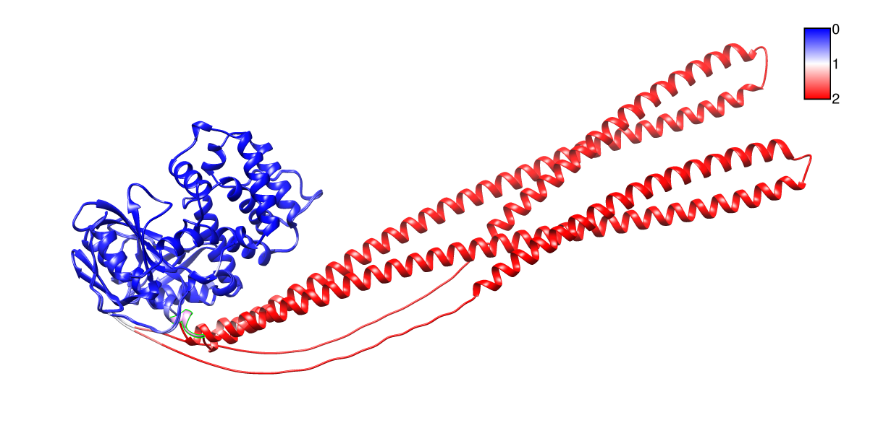

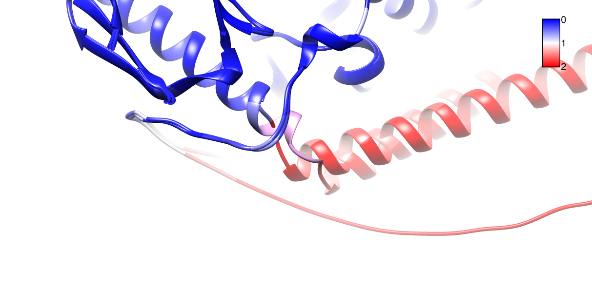


**(Å)**

**(Å)**


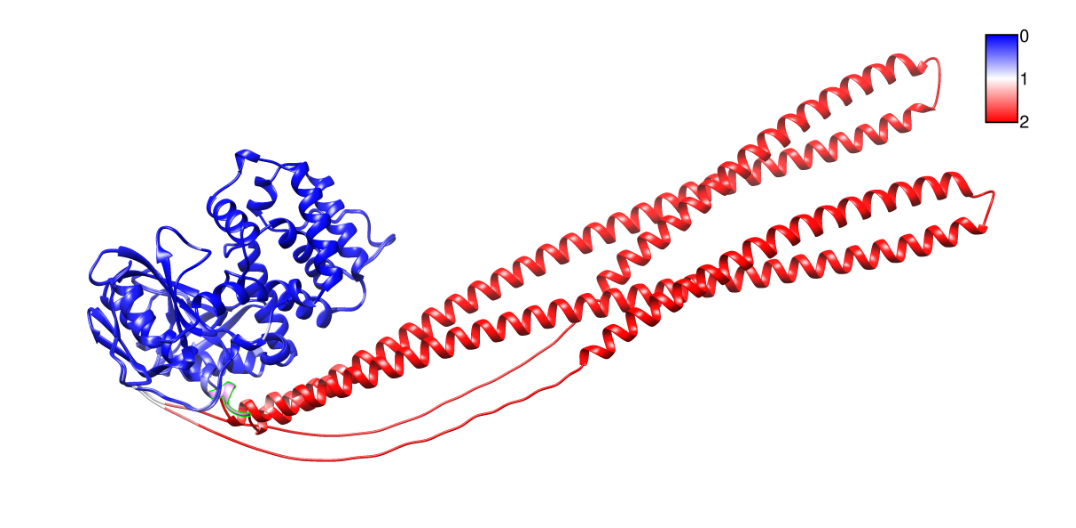


**Figure S4. Deletion of the RRRK_294-297_ amino acids of the NLS (Moe-DNLS) cause only minor local structural changes in the FERM domain.** However, it might affect the position of the coiled-coil structure (alpha-helix region). **(A-B)** shows RMSD calculated for all atoms. Scale bars refer to Ångström.

**A.**


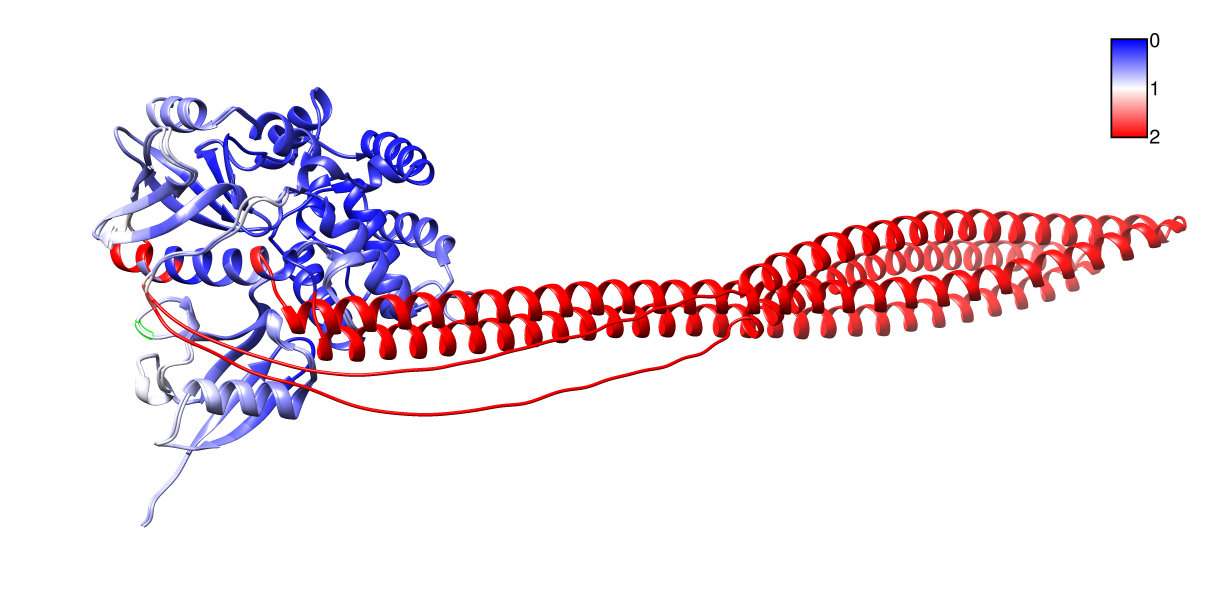


**(Å)**


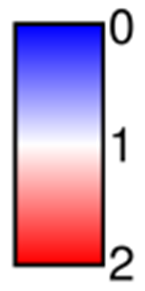

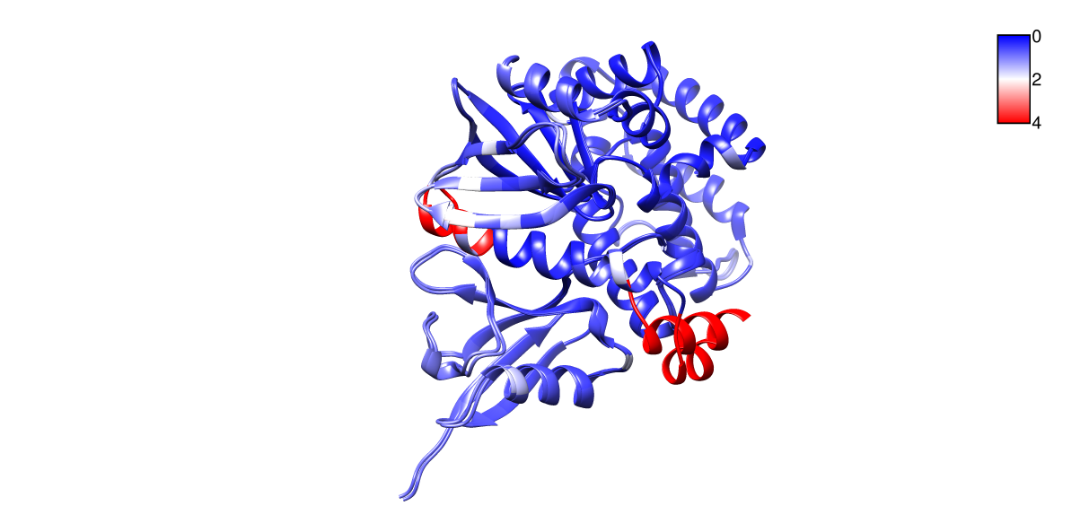

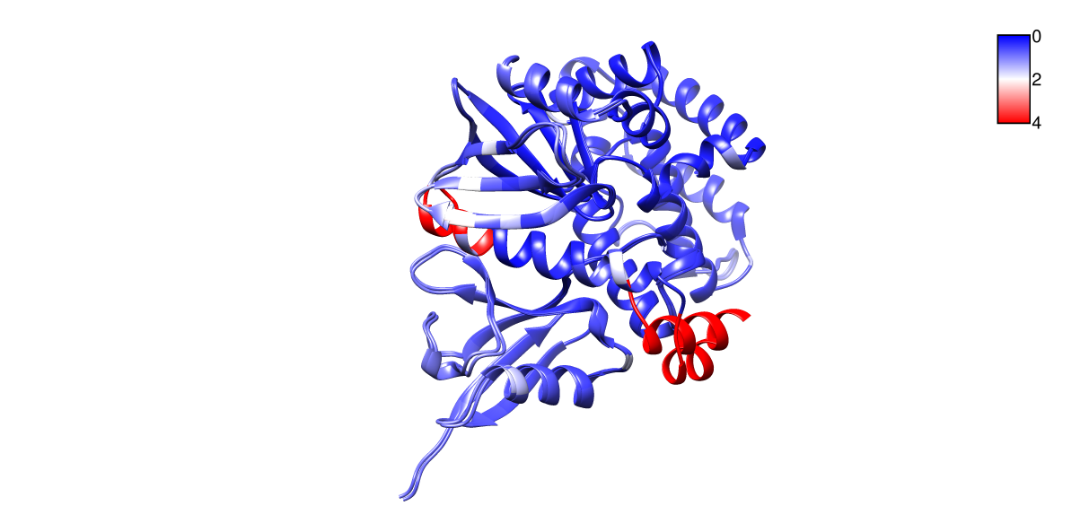


**C.**

**B.**

**(Å)**

**(Å)**


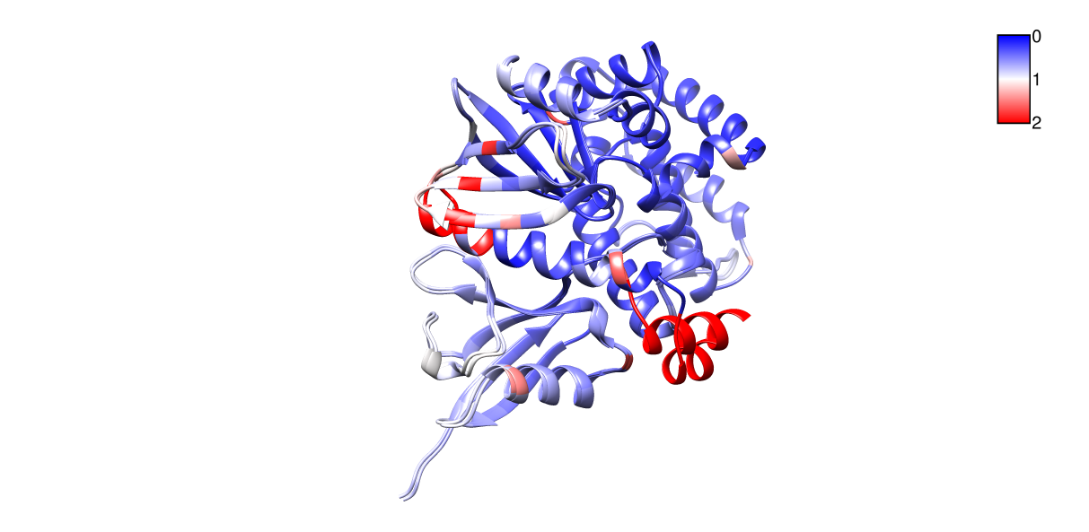


**Figure S5. Comparison of AlphaFold2 models of KR_279-280_ and RRRK_294-297_ double mutant, and wt moesin proteins. (A-C)** RMSD calculated for all atoms. **(B-C)** The FERM and CTD domains are shown without the alpha-helix region. Notice the different scales. Scale bars refer to Ångström.
